# Molecular Equilibrist: The Small Heat Shock Protein IbpA from Mycoplasma

**DOI:** 10.3390/biom16060891

**Published:** 2026-06-17

**Authors:** Innokentii E. Vishnyakov, Alexey D. Vedyaykin

**Affiliations:** 1 Institute of Cytology, Russian Academy of Sciences, Tikhoretsky Ave. 4, 194064 Saint-Petersburg, Russia; 2 Institute of Biomedical Systems and Biotechnology, Peter the Great St. Petersburg Polytechnic University, Polytechnicheskaya 29, 195251 Saint-Petersburg, Russia; vedyajkin_ad@spbstu.ru

**Keywords:** small heat shock proteins, mycoplasma, fibrils, globules, cold shock, heat shock

## Abstract

Small heat shock proteins (sHSPs) serve as “first aid” stress-response proteins in both eukaryotes and prokaryotes. Their holdase activity enables binding to partially denatured proteins, maintaining them in a folding-competent state under stress. The sHSP IbpA from the mycoplasma *Acholeplasma laidlawii* is a unique member of its family, combining the functions of two *Escherichia coli* sHSPs that typically act in tandem. In this study, we demonstrate for the first time that IbpA forms distinct supramolecular structures under contrasting temperature stresses in crowded environments without any artificial truncations or mutations at the protein termini. Upon cooling, IbpA in vitro forms long fibril bundles, whereas heating induces the formation of large, rounded agglomerates. At the temperature optimal for culture growth, the protein exists as a mixture of short fibrils and small globules, with the latter predominating. IbpA’s cellular localization mirrors in vitro properties, with an increased proportion of surface-associated proteins among the sHSP partners during cold shock. We also report, for the first time, a rapid and reversible transition of IbpA to a fibrillar form in response to cold. We propose hypotheses regarding potential roles of IbpA in the mycoplasma cell. IbpA from *A. laidlawii* appears to act as a “molecular equilibrist,” protecting the cell against damage under opposing stresses, though the precise mechanism of its action during cold shock remains to be elucidated.

## 1. Introduction

Small heat shock proteins (sHSPs, HSP20) are ATP-independent chaperones that are widely distributed in nature and found in the cells of most eukaryotes and prokaryotes, serving as a first line of defense against stress [1]. Their primary mechanism is believed to involve binding to unfolded, misfolded, or partially denatured proteins early during stress, thereby maintaining them in a folding-competent state for subsequent refolding by ATP-dependent chaperones such as HSP70 and HSP110 [2]. Initially, sHSPs were characterized as heat shock proteins that protect cellular proteins from irreversible aggregation at elevated temperatures [3,4]. However, the understanding of their cellular functions has since expanded significantly.

Currently, evidence indicates that sHSP production increases, their oligomeric structures reorganize, and they protect proteins under various stress conditions, including oxidative stress [5], pH shifts [6], exposure to metal ions [7], and mechanical stress [8]. Recent studies have begun to report the role of sHSPs in enhancing organismal survival under cold stress; however, the mechanisms underlying their function in these conditions remain unclear [9,10,11,12].

In their monomeric form, sHSPs range in size from 12 to 43 kDa [13]; however, they lack chaperone (holdase) activity in this state. The supramolecular assemblies of various sHSPs vary widely in size, ranging from dimers [14] to 12, 16, 24, 32-mers [15], and even larger complexes of 2–3 MDa [16]. It has been suggested that the dimeric interface within these oligomers is essential for their chaperone-like function [17].

Canonical sHSPs comprise an alpha-crystallin domain flanked by adjacent N-terminal and C-terminal regions [18]. The N-terminal domain typically includes a conserved WDPF motif, which plays a key role in protein oligomerization and the assembly of large globular structures [19]. Additionally, the conserved (I/V)*X*(I/V) motif in the short C-terminal domain interacts with the central alpha-crystallin domain, affecting both the oligomerization process and the chaperone activity of sHSPs [20].

Temperature fluctuations or other stress factors that induce protein denaturation are believed to alter the oligomeric state of sHSPs, and to lead to the exposure of their hydrophobic regions. These exposed hydrophobic areas then interact with the hydrophobic regions of partially denatured substrate proteins of sHSPs [21,22]. Through this interaction, sHSPs form large aggregates that remain poised for subsequent processing by ATP-dependent chaperones and proteases once the stress conditions subside [23].

In both eukaryotic and prokaryotic cells, sHSPs often function cooperatively rather than individually. Human cells contain at least ten different sHSPs that interact with one another in various ratios and combinations [24], enabling precise regulation of the multichaperone network. Similarly, in the bacterial cell of *Escherichia coli*, two sHSP homologues, IbpA and IbpB, operate in close cooperation [16]. *E. coli* sHSPs are distinctive members of the sHSP family. IbpA in *E. coli* is active in its fibrillar form, where it binds to target proteins and preserves them in a folding-competent state until ATP-dependent chaperones DnaK (HSP70) and ClpB (HSP100) can act [25]. However, IbpA alone cannot efficiently transfer substrates to the HSP70-HSP100 system for proper folding; this requires interaction with a second component, IbpB, which predominantly exists as globular structures in the cell. Together, IbpA and IbpB form a unique system wherein two sHSP components assemble into distinct supramolecular structures, each mediating different functions toward a shared goal [26].

The plant pathogen *Acholeplasma laidlawii* contains a single sHSP homolog, the IbpA protein [27]. Recent research has shown that this IbpA exhibits unique properties by combining the functions of both *E. coli* sHSPs, IbpA and IbpB: its C-terminal domain is responsible for forming the fibrillar sHSP structure and binding substrates, while the N-terminal domain, which includes a conserved dual WDPF-like motif, drives the formation of globular structures [28]. These N- and C-terminal regions competitively regulate the oligomerization pattern and chaperone-like activity of IbpA. The efficiency of the mycoplasma multi-chaperone system relies on IbpA’s ability to form both globular and fibrillar supramolecular structures, effectively merging the roles of IbpA and IbpB in transferring substrate proteins to the HSP70-HSP100 system [29].

In this work, we advanced our understanding of the factors driving that shift in the oligomerization pattern of the sHSP IbpA in the *A. laidlawii* cell, as well as proposed the additional physiological roles that the supramolecular structures formed by IbpA may play during cold and heat stress.

## 2. Materials and Methods

### 2.1. Bacterial Strains and Media

*Acholeplasma laidlawii* PG8 cells (from the collection of the Institute of Cytology, Russian Academy of Sciences) were thawed at room temperature and transferred into Mycoplasma broth base liquid medium (OXOID, Basingstoke, Hampshire, UK) supplemented with Supplement G (OXOID, UK) and 1% sterile glucose. Phenol red was added to the medium as a pH indicator until it developed a crimson color (final concentration ~0.04%). The mycoplasma culture was incubated at 37 °C for approximately 24 h without shaking in sterile test tubes (Falcon 15 mL, Eppendorf, Hamburg, Germany) sealed with tightly screwed caps. Bacterial growth was monitored by a color change in the medium from crimson to straw-yellow. After about 24 h, a 1:10 inoculum was transferred to fresh liquid mycoplasma growth medium, and the culture was further incubated at 37 °C until the medium again turned straw-yellow, typically within 24 h or slightly longer.

### 2.2. Protein Purification and Storage

The recombinant IbpA protein of *A. laidlawii* used in this study was produced in *E. coli* cells and purified in preparative quantities during a previous investigation [27]. Immediately following purification, the protein was dialyzed against 1× PBS and stored in a lyophilized form. Prior to the experiments, the lyophilized protein was reconstituted by adding 1 mL of 1× PBS buffer to achieve a final concentration of 3 mg/mL. After dissolving the lyophilisates in the buffer, large protein aggregates were removed by centrifugation at 13,000 rpm for 10 min using a MiniSpin centrifuge (Eppendorf, Hamburg, Germany).

### 2.3. Temperature Conditions

For the in vitro temperature experiment, IbpA was mixed in 1× PBS with 10% PEG_4000_ (0.8 mg/mL) and supplemented with 5 mM ATP or GTP, and the mixture was incubated for 30 min at different temperatures (4 °C, 30 °C, 37 °C, and 42 °C). After incubation, the mixtures were carefully pipetted, and 5 μL of each sample were collected on nickel grids for negative staining in triplicate.

Before the in vivo temperature experiment, the *A. laidlawii* culture, grown at the optimal temperature of 37 °C, was divided into three equal parts. One part was maintained at the optimal temperature, while the second part was subjected to heat shock by increasing the temperature from 37 °C to 42 °C for 90 min. Following this, the bacteria were returned to 37 °C and incubated for an additional 90 min. Previous studies have shown that these conditions promote maximal accumulation of the small heat shock protein IbpA in the mycoplasma cell [27,30]. The third part of the culture was cooled to 4 °C and incubated for 90 min to match the duration of the heat shock treatment. Subsequently, these bacteria were also returned to 37 °C for a further 90 min of incubation.

### 2.4. Negative Staining

Protein samples were negatively stained on grids following the procedures described in [28,29]. Briefly, nickel grids (400 mesh, Electron Microscopy Sciences, Fort Washington, PA, USA) were coated with a 2% collodion solution in amyl acetate (Electron Microscopy Sciences, USA) to form a film and allowed to dry overnight at room temperature. The grids were then exposed to UV light for 1 min, after which 5 µL of the protein solution in the appropriate buffer with additives was applied for 15 s and subsequently removed using filter paper. This was followed by the addition of 5 µL gadolinium triacetate (Uranyl Acetate Alternative, Ted Pella, Redding, CA, USA) for 15 s, with the stain agent similarly removed by filter paper. Samples were visualized using a Libra 120 electron microscope (Carl Zeiss, Jena, Germany) at 10,000× magnification.

### 2.5. Electron Microscopy

*A. laidlawii* cells, after the temperature experiment, were chemically fixed directly in the medium using glutaraldehyde at a final concentration of 2.5% for 30 min at room temperature. The cells were then collected by centrifugation at 10,000 rpm for 5 min at room temperature. The resulting cell pellets were postfixed in 1% osmium tetroxide (the solution was prepared by diluting 4% osmium tetroxide, Electron Microscopy Sciences, Fort Washington, PA, USA, with sterile distilled water) for 30 min at room temperature and embedded in an epon–araldite mixture [31]. The resin was polymerized at 60 °C for 2 days. Ultrathin sections were cut with a diamond knife (Diatome, Nidau, Switzerland) using an Ultratome III 8800 ultramicrotome (LKB, Bromma, Sweden) and transferred to nickel grids (400 mesh, Merck, Darmstadt, Germany) coated with collodion. Sections were stained with gadolinium triacetate (Uranyl Acetate Alternative, Ted Pella, Redding, CA, USA). Electron microscopy was performed using a Libra 120 electron microscope (Carl Zeiss, Jena, Germany) at magnifications of 8000× to 16,000×.

### 2.6. Immune-Electron Microscopy

Mycoplasma cells were fixed directly in the growth medium by adding formaldehyde to a final concentration of 2% and glutaraldehyde to 0.1%, followed by incubation for 1 h at room temperature. After fixation, the cells were collected by centrifugation at 10,000 rpm for 5 min at room temperature. The pellets were dehydrated through a graded ethanol series (70% for 15 min, then 96% for 15 min) and subsequently infiltrated with LR-White acrylic resin (Polysciences, Warrington, PA, USA) in ethanol at ratios of 1:3 for 2 h at room temperature, 1:1 overnight at 4 °C, and 3:1 for 2 h at room temperature. Following an additional 2-h incubation in pure LR-White resin at room temperature, the pellets were transferred to gelatin capsules for embedding. Resin polymerization was carried out at 50–52 °C for 2 days. Ultrathin sections were prepared using an Ultratome III 8800 ultramicrotome (LKB, Bromma, Sweden) and placed on UV-discharged collodion-coated nickel grids (400 mesh, Merck, Darmstadt, Germany). The sections were incubated with custom-made anti-IbpA antibodies [27] diluted 1:50 in 1× PBS containing 0.1% BSA. Instead of secondary antibodies, a protein A conjugate with 15 nm colloidal gold particles (EY Laboratories, San Mateo, CA, USA) was applied. Sections were stained with gadolinium triacetate (Uranyl Acetate Alternative, Ted Pella, Redding, CA, USA) for 15 min at room temperature. Electron microscopy was performed using a Libra 120 microscope (Carl Zeiss, Jena, Germany). The distribution of colloidal gold particles within the mycoplasma cells was quantified by counting particles in five fields of view for each temperature condition.

### 2.7. Light Scattering

The in vitro formation of large supramolecular structures by the IbpA protein was monitored using 90° light scattering. Acrylic cuvettes containing a mixture of protein in 1× PBS (0.8 mg/mL) supplemented with 10% PEG_4000_ were placed in a Varian Cary Eclipse (Agilent Technologies, Penang, Malaysia) fluorescence spectrometer (settings: excitation wavelength 350 nm; emission wavelength 350 nm; excitation slit 5 nm; emission slit 5 nm; detector voltage low) maintained at 30 °C. After confirming a stable scattering signal, GTP was added to a final concentration of 5 mM. Immediately following GTP addition, the samples were incubated directly in the cuvettes for 10 min at the designated temperatures (4 °C, 30 °C, 37 °C, and 42 °C). Subsequently, the cuvettes were returned to the spectrometer chamber, and spectra were recorded for several minutes. This cycle was repeated four times over the course of one hour for each sample.

## 3. Results

### 3.1. In Vitro, the Small Heat Shock Protein IbpA from A. laidlawii Forms Distinct Large Supramolecular Structures That Vary According to the Temperature

Previous studies have highlighted the unique properties of the sHSP IbpA from *A. laidlawii* compared to other bacterial sHSPs [28,29,32]. To date, IbpA from *A. laidlawii* is the only known member of this protein family in which a competitive interplay between the N- and C-terminal domains governs the shift of its quaternary structure between fibrillar and globular forms, thereby providing a molecular mechanism for functional regulation [29]. In this study, we aimed to further understand the physiological conditions that may trigger this structural shift within the cell.

First, we aimed to investigate the effect of temperature on the formation of IbpA fibrils or globules. It is important to note that previous size-exclusion chromatography data [28] indicated that temperature did not alter the protein peak profiles during column extraction, suggesting that temperature might not influence the ratio of fibrils to globules. However, the cellular environment where the proteins’ function differs significantly from the conditions in the extraction buffer, which could impact this behavior.

To better mimic the conditions of the bacterial cytoplasm, we added the crowding agent PEG_4000_ to the protein suspension. Additionally, we examined the effects of ATP and GTP on the formation of IbpA fibrils or globules, despite sHSPs not being ATP-dependent chaperones [22]. It has been shown for ATP to induce conformational changes in sHSPs, facilitating the release of substrate proteins from sHSP-substrate complexes for subsequent refolding by the HSP70-HSP100 chaperone system [33]. Furthermore, evidence shows that ATP and GTP affect the formation of stress granules through phase separation [34,35], and the composition of these biomolecular condensates formed during stress includes small heat shock proteins, which act as modulators of condensate dynamics [36]. Protein samples were visualized using negative staining.

**Figure 1:**
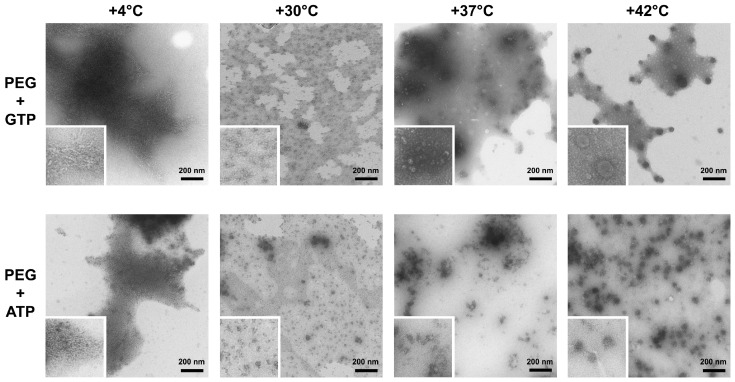
In vitro temperature-dependent behavior of the IbpA protein from mycoplasma *A. laidlawii* under crowding conditions. Negative staining. GTP and ATP denote the nucleotides added to the protein suspension. The electron-dense material connecting the IbpA structures likely reflects specific dye distribution on the grids, resulting from the presence of the crowding agent PEG_4000_. Inserts: Slightly enlarged images of the most characteristic supramolecular structures of IbpA formed at a given temperature in the presence of the selected nucleotide. Scale bar: 200 nm. Original images in Supplementary Materials.

We demonstrated that under crowded conditions, IbpA from *A. laidlawii* forms distinct large supramolecular structures depending on the temperature. At 4 °C, regardless of the presence of ATP or GTP, IbpA appeared as large bundles of fibrils under the microscope (Figure 1, 4 °C). At optimal mycoplasma growth temperatures (30 °C and 37 °C), both short fibrils and small protein globules were observed, with a marked predominance of globular forms, and an increasing tendency for globules and both thin and thicker fibrils to grow larger as the temperature increased. When heated to 42 °C (Figure 1, 42 °C), simulating mild heat shock, IbpA primarily formed large, rounded aggregates approximately 50 nm in diameter. Notably, on average, the aggregates become larger when GTP is added compared to when ATP is added. This is the sole structural difference in the aggregates depending on the type of nucleotide present.

Thus, temperature conditions emerge as a key factor influencing the formation of fibrillar or globular supramolecular forms of IbpA in *A. laidlawii* cells. Notably, this study provides the first evidence that sHSP can transform into a fibrillar form in response to a strong decrease in temperature.

### 3.2. The Behavior of the IbpA Protein In Situ May Reflect the Patterns and Structural Changes We Observed In Vitro

A 2012 study [27] first demonstrated that under heat stress, IbpA in *A. laidlawii* is not randomly distributed within the mycoplasma cell but instead binds to specific cellular structures. Notably, among these structures over 60% of the labeled IbpA was detected on electron-dense compartments known as “granular bodies” [37].

**Figure 2:**
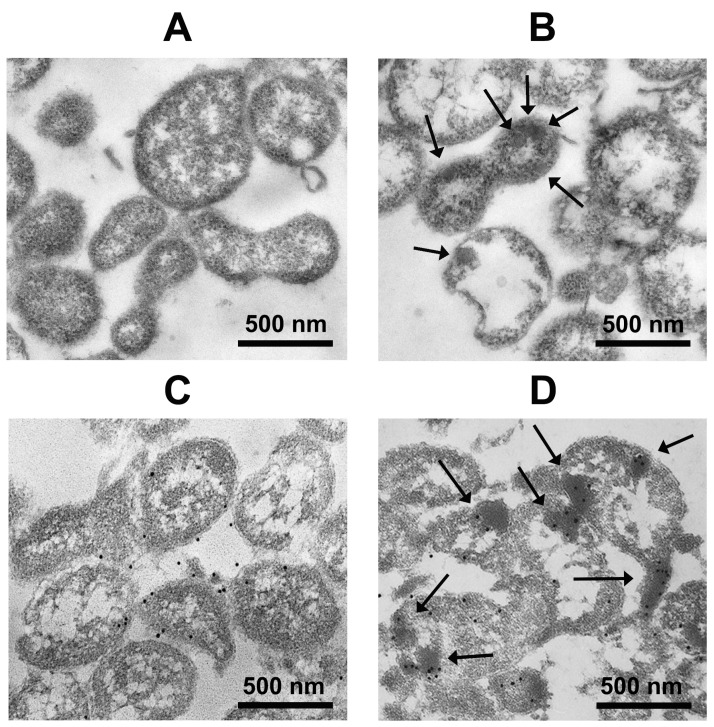
Association of IbpA with the so-called “granular bodies” in the *A. laidlawii* cell during heat shock. (**A**) morphology of bacterial cells at the optimal cultivation temperature (37 °C); (**B**) morphology after heat shock exposure (42 °C); (**C**) localization of IbpA in the mycoplasma cell at 37 °C; (**D**) localization of IbpA at 42 °C. The “granular bodies” of *A. laidlawii* are indicated by arrows. Colloidal gold particles (15 nm diameter) are cross-linked with protein A, which binds to the primary rabbit antibodies against IbpA from *A. laidlawii*. Scale bar: 500 nm. Original images in Supplementary Materials.

Here, we confirm the preferential association of colloidal gold particles conjugated to protein A (which binds to primary rabbit antibodies against IbpA from *A. laidlawii*) with these electron-dense compartments during heat shock (Figure 2). Interestingly, the size of the “granular bodies” (50–100 nm in diameter) closely matches the size of IbpA aggregates formed in vitro at 42 °C in the presence of PEG_4000_.

**Figure 3:**
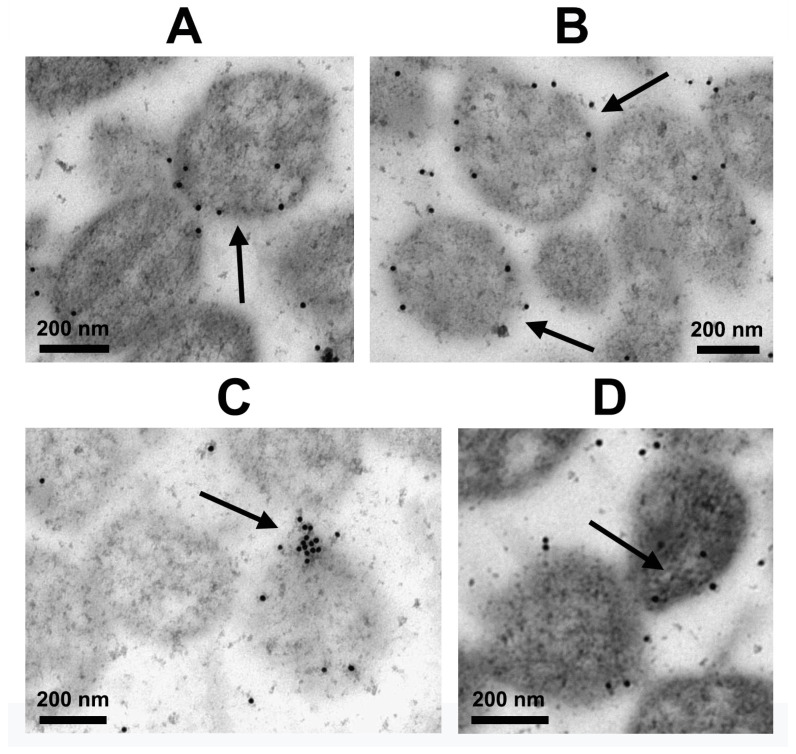
Immunolocalization of the IbpA protein in *A. laidlawii* cells exposed to cold shock (4 °C). (**A**,**B**) predominant localization of the sHSP near the cell surface (79.8 ± 7.12%); (**C**,**D**) section planes passing through structured cell areas where the specific label is concentrated. Arrows indicate cells with the clearest localization of colloidal gold particles (electron-dense spheres approximately 15 nm in diameter). Scale bar: 200 nm. Original images in Supplementary Materials.

Upon cold shock (4 °C), IbpA *A. laidlawii* is predominantly localized at the cell periphery, near the membrane (Figure 3A,B). In some instances, “label bundles” are observed, where the labeling appears as beads strung along certain structures that are not clearly visible due to the embedding method used (Figure 3C). Occasionally, distinctive structuring within the mycoplasma cells becomes apparent (Figure 3D), potentially when the section plane is closer to the cell surface.

We roughly estimated the percentage of colloidal gold particles located near the mycoplasma cell surface at different temperatures by counting in five fields of view per temperature. The analysis considered label localization at the cell periphery, without distinguishing specific membrane association. Under heat shock (42 °C), 50.8 ± 3.19% of the label was near the cell surface. This percentage increased to 60.6 ± 8.17% at the optimal growth temperature (30 °C) and reached a maximum of 79.8 ± 7.12% under cold shock (4 °C).

Thus, it is possible that IbpA fibrils or fibril bundles also form in situ under cold shock conditions, shifting away from their direct chaperone function to potentially serve an additional structural role (for example, helping to protect *A. laidlawii* cells, which lack a cell wall, from cold stress).

### 3.3. Dynamics of the Formation of Large Supramolecular Structures of IbpA

Based on the data obtained, we investigated the speed and reversibility of the transition between different supramolecular forms of IbpA in response to temperature changes. To do this, we employed light scattering measurements. PEG_4000_ was added to the IbpA protein solution in 1× PBS, and spectral recording began immediately. Samples were then incubated directly in cuvettes at various temperatures for 10 min each: in a refrigerator (4 °C), in thermostats (37 °C and 42 °C), or in the spectrophotometer chamber, which kept a constant temperature of 30 °C. After each 10-min incubation, all samples were returned to the spectrophotometer chamber for continued recording over several minutes. This cycle was repeated three more times, totaling four cycles within one hour.

**Figure 4:**
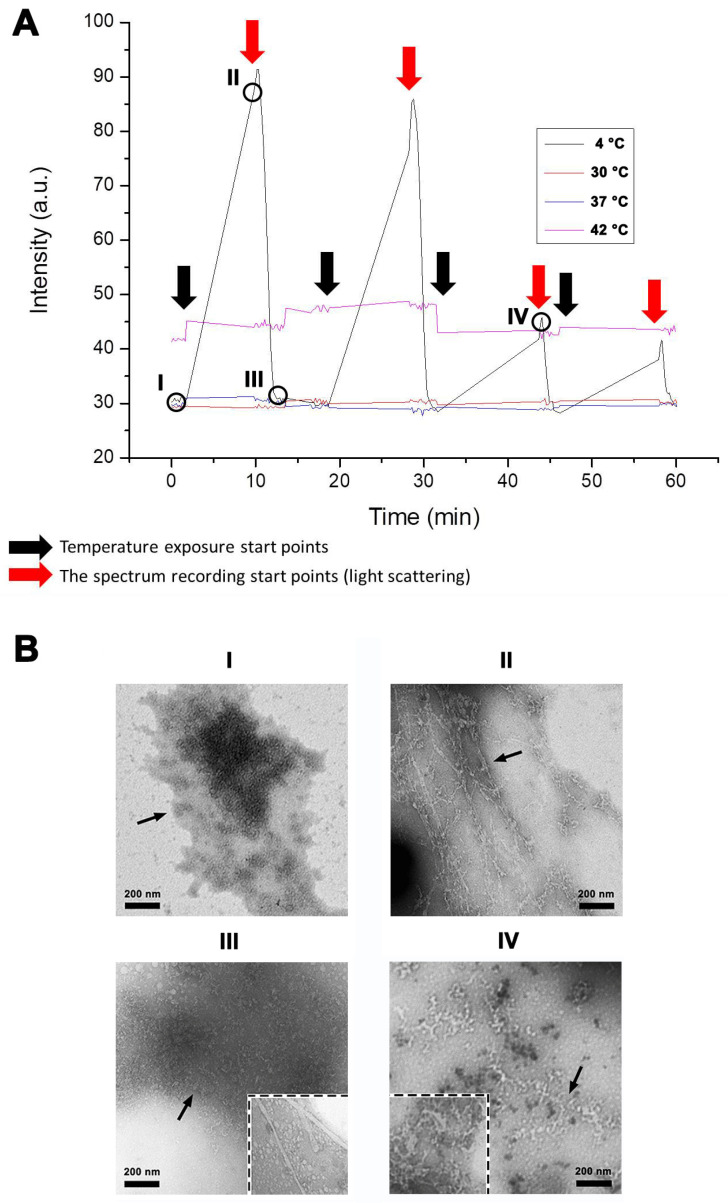
IbpA response to short-term repeated temperature exposures. Each IbpA incubation cycle involved 10 min at 4 °C, 30 °C, 37 °C, or 42 °C, followed by recording the light scattering signal in a spectrophotometer chamber maintained 30 °C for several minutes. This cycle was repeated up to four times within an hour. (**A**) light scattering curves; (**B**) electron micrographs of IbpA supramolecular structures (indicated by arrows). Numbers of the electron micrographs I–IV on (**B**) correspond to the sampling points indicated by black circles on the light scattering curve “4 °C” on (**A**). Inserts (highlighted with a dashed line): III—an example of the rare long strands of IbpA; IV—an example of fibril bundles of IbpA. Original images in Supplementary Materials.

The experiment demonstrated that the turbidity of the IbpA protein solution increased sharply after just 10 min at 4 °C (Figure 4A). When the solution was then rapidly reheated to 30 °C in the spectrophotometer chamber, the turbidity reversed quickly, and the solution became clear in under 2–3 min. This cycle repeated similarly in the second run. In the third and fourth cycles, turbidity still increased but by a smaller amount—less than half of that observed in the first two cycles—and showed a decreasing trend. Despite these changes, the protein solution consistently returned to a clear state upon heating each time.

At 30 °C and 37 °C, the solution’s turbidity remained essentially stable, which aligns with the previously established characteristics of the sHSP [28,29]. At 42 °C, turbidity increased compared to the optimal mycoplasma growth temperatures, although it did not reach the high levels seen at 4 °C. Additionally, no abrupt changes in turbidity were observed between cycles at this temperature.

To investigate the cause of turbidity changes in the solution during cooling and subsequent heating to 30 °C, samples were collected at specific time points, applied to nickel grids, negatively stained, and examined by transmission electron microscopy (Figure 4B).

This approach showed that at point I (30 °C), IbpA is predominantly found as small globular structures in the mixture. By the end of the first exposure to 4 °C (point II), large branching fibrils and fibril bundles become clearly visible under the microscope. When the sample is returned to the spectrophotometer chamber at 30 °C (point III), these fibrils partially break down, leaving only a few long strands (shown in the insert), while the remaining protein appears as small globules or short fibrils. During the third exposure cycle at 4 °C (point IV), when the turbidity increase begins to slow, branching fibrils and fibril bundles (shown in the insert) are again observed, though they are noticeably shorter than those seen during the initial cooling.

Thus, the fastest and reversible transition between different forms of IbpA indeed occurs during cooling, driven by the rapid formation of fibrils and fibril bundles, which causes the marked increase in turbidity. The solution’s return to transparency is due to the rapid disintegration of these fibrils. The protein can endure multiple repeated cycles of this process, but its fibrils gradually become shorter over time.

## 4. Discussion

The results of this study suggest that the transition between fibrillar and globular supramolecular structures of the sHSP IbpA from *A. laidlawii* can occur within the mycoplasma cell and may play a certain role. For the first time, we demonstrated that this transition is temperature-dependent (Figure 1) and, in the case of short-term cold exposure, rapid and reversible (Figure 4). Notably, the in vitro findings from TEM with negative staining and light scattering analysis, align well with the electron microscopy results (Figure 2 and Figure 3) and previously obtained proteomic data [38].

In a 2016 study [38], it was shown that the set of IbpA target proteins varies depending on the temperature to which mycoplasma cells are exposed. At 4 °C, 308 proteins co-eluted with sHSPs, whereas at 46 °C, 464 proteins co-eluted. Among these presumed sHSP targets, 68 proteins interacted exclusively with IbpA in the cold, while 224 proteins were specific targets at the elevated temperature. Additionally, 240 proteins co-eluted with IbpA at both 4 °C and 46 °C.

Among the 68 unique proteins co-eluting with IbpA during cooling, 25 were identified as either containing transmembrane domains or being characterized as bacterial cell surface proteins, integral membrane proteins, surface-anchored sensors involved in signal transduction, or components of transport systems that naturally reside on the mycoplasma cell surface. This means that 36.8% of the proteins interacting with IbpA at 4 °C are associated with the mycoplasma cell surface. At 46 °C, the number of IbpA target proteins localized near the mycoplasma cell surface increases to 35. However, these proteins represent only 15.6% of the total 224 proteins co-eluting with IbpA at this elevated temperature. Of the 240 proteins co-eluting with IbpA at both 4 °C and 46 °C, 59 were classified as bacterial cell surface proteins based on various criteria. This accounts for 24.5% of all the “temperature-independent” sHSP target proteins.

To obtain a precise understanding, of course, the cellular abundance of each unique protein must be considered. However, there is a clear trend showing an increase in the percentage of the sHSP substrate proteins associated with the cell surface as the temperature decreases. This trend aligns well with the immune-electron microscopy data obtained in the present work (see Section 3.2).

Regarding the impact of sHSPs on organism survival during cold shock, current research has only begun to explore the potential mechanisms of these chaperones, with studies so far providing only a preliminary understanding [9,10,11,12]. For example, transcripts of sHSP 17.4-CII and sHSP 23.8-M were found to accumulate significantly in chilling-tolerant tomato fruits [9]. The expression of sHSP genes in *Haemaphysalis longicornis* is influenced by the severity and duration of cold exposure, with RNA interference reducing tick survival [11]. In the cigarette beetle, *Lasioderma serricorne*, three LsHsp genes were notably upregulated in response to both heat and cold stress [10]. The SmHsp26.7 gene in *Sitodiplosis mosellana* showed higher expression in winter compared to summer, and short-term cold shock triggered transcriptional upregulation of sHSP genes in overwintering larvae [12]. Similarly, Cshsp22.9b and Cshsp24.3 genes in the rice pest *Chilo suppressalis* were upregulated by temperature extremes [39]. Overall, research on sHSPs during cold shock has largely been limited to transcriptional studies. It is important to note that proteins in the cell can undergo cold denaturation [40,41,42,43,44]; however, it remains unclear whether the way sHSPs interact with their targets during cold stress is similar to their mechanisms during heat shock or other stresses.

In the case of *A. laidlawii*, the adaptive response to cold shock mediated by sHSP likely involves a mechanism distinct from the typical exposure of hydrophobic regions during stress and is associated with the formation of different supramolecular structures (Figure 1 and Figure 4). It is known that IbpA *E. coli* also forms fibrils, which are thought to serve as an inactive storage form [45]. In contrast, we previously demonstrated that IbpA *A. laidlawii* remains active as a chaperone even in its fibrillar state [28]. The active, rather than passive, role of IbpA fibrils in protecting *A. laidlawii* cells from cold stress is supported by changes in the pattern of proteins interacting with IbpA under contrasting temperature conditions [38]. Along with evidence of increased expression of small heat shock protein genes in certain higher eukaryotes under cold stress [9,10,11,12], and our new findings outlined above, this suggests that IbpA may play an active protective role during cooling. However, until more precise data are available, the physiological significance of IbpA fibrillation in response to cold remains speculative.

For example, in addition to protecting a number of proteins from cold denaturation, rapid fibrillization of IbpA at low temperatures may play mechanistic, structural role in the mycoplasma cell. The interaction of fibrillar IbpA with surface-associated proteins near the cytoplasmic membrane—the sole natural boundary of the mycoplasma cell, which lacks a cell wall—during short-term cold shock could facilitate the rapid formation of a submembrane protective “protein stress shell.” This “shell” could prevent membrane damage during abrupt temperature changes, such as transitions through freezing point, thereby providing an additional mechanism to enhance mycoplasma cell survival. However, this still requires confirmation through further in vivo experiments.

At 42 °C, the sHSP is primarily associated with the mycoplasma’s “granular bodies,” which may represent inclusion bodies [46,47,48], stress granules [49], or co-aggregates of partially denatured proteins with the sHSP [50]. Notably, the size of large IbpA globules formed in vitro at elevated temperatures closely matches that of the mycoplasma’s in situ “granular bodies”. However, their composition is understandably quite different, since in vitro experiments involve isolated and purified IbpA protein, whereas in the cell, the sHSP interacts with a diverse pool of target proteins.

Emerging evidence suggests that sHSPs are components of stress granules in cells across various organisms and play a crucial role in their function [36]. Additionally, GTP and ATP influence the formation of these biomolecular condensates through liquid–liquid phase separation [34,35]. Further experiments are needed to determine whether “granular bodies” form in *A. laidlawii* cells under heat shock by a similar mechanism. Regardless, the processes by which IbpA forms large globular structures versus long, branched fibrils—and their respective roles—are likely distinct.

Additionally, we observed certain differences between the effects of ATP and GTP on the supramolecular structures formed by IbpA at 42 °C (Figure 1). When ATP was added to the mixture instead of GTP, large IbpA aggregates became more compact, decreasing slightly in size. In NtHSP18P (HSP18), a cytosolic class I small heat-shock protein from tobacco pollen grains, ATP has been shown to promote a more compact oligomer structure and reduce chaperone activity [51]. It is possible that IbpA *A. laidlawii* at elevated temperatures behaves similarly in the presence of ATP.

At optimal temperatures for *A. laidlawii* culture growth, we observed—a finding consistent with our previous studies [28,29]—a mixture of small IbpA globules and short fibrils, with the globules predominating. These sHSP supramolecular structures may represent transitional forms of the protein.

**Figure 5:**
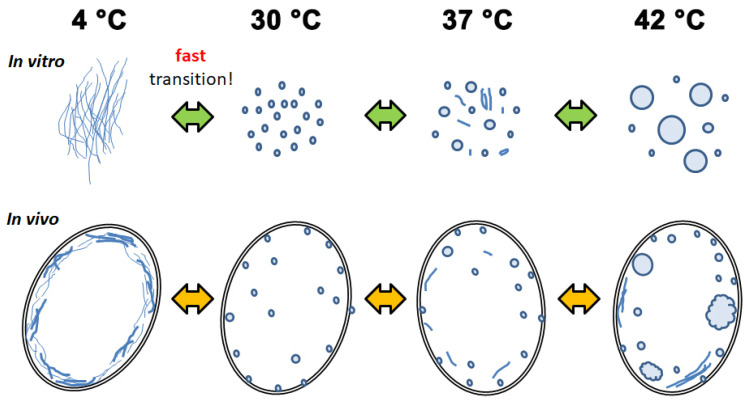
A highly simplified scheme illustrating the formation of supramolecular structures by the IbpA protein. The scheme depicts their reversible transitions both in vitro and in vivo in response to environmental temperature changes. Original images in Supplementary Materials.

Considering all of the above, we propose a highly simplified and preliminary model illustrating the formation and interconversion of IbpA supramolecular structures in *A. laidlawii* cells in response to environmental temperature changes (Figure 5).

## 5. Conclusions

Bullet points of the investigation are as follows:

- The shift between fibrillar and globular supramolecular structures of the mycoplasma sHSP IbpA, in particular, is temperature-dependent;
- IbpA localization patterns in *A. laidlawii* cells differ during heat and cold shock;
- The formation of the sHSP fibrils in the cold is rapid and reversible;
- The sHSP large aggregates in the heat are more stable;
- The proportion of mycoplasma cell surface-associated proteins interacting with IbpA increases as temperature decreases.

Many researchers have attempted to extend the understanding of sHSP mechanisms at elevated temperatures to other stress conditions. We hypothesize that sHSPs can fulfill multiple roles within the cell simultaneously, adopting distinct conformations and forming various supramolecular structures depending on the surrounding environmental conditions. For example, the sHSP IbpA from *A. laidlawii* may act as a “molecular equilibrist,” helping to maintain mycoplasma cell integrity under contrasting temperature conditions. Its mechanism of action likely differs between heat shock and cold stress, reflecting significant changes in its supramolecular structures. Based on our experimental findings and theoretical insights, we suggest investigating properties of different sHSPs in other micro- and macroorganisms in a similar way. Such studies may reveal answers to the diverse and still enigmatic functions of sHSPs within the cell.

## Data Availability

The original contributions presented in this study are included in the article. Further inquiries can be directed to the corresponding author.

## Supplementary Materials

The following supporting information can be downloaded at: https://www.mdpi.com/article/10.3390/biom16060891/s1, TEM and IEM original images.

## Abbreviations

The following abbreviations are used in this manuscript:

sHSP(s)	Small heat shock protein(s)
ATP	Adenosine triphosphate
GTP	Guanosine triphosphate
PEG	Polyethylene glycol
PBS	Phosphate-buffered saline
