# Supplementary figures and images for: Molecular Equilibrist: The Small Heat Shock Protein IbpA from Mycoplasma
Source: Biomolecules. 2026 Jun 17;16(6):891. doi: 10.3390/biom16060891 (PMC13297169; doi:10.3390/biom16060891)

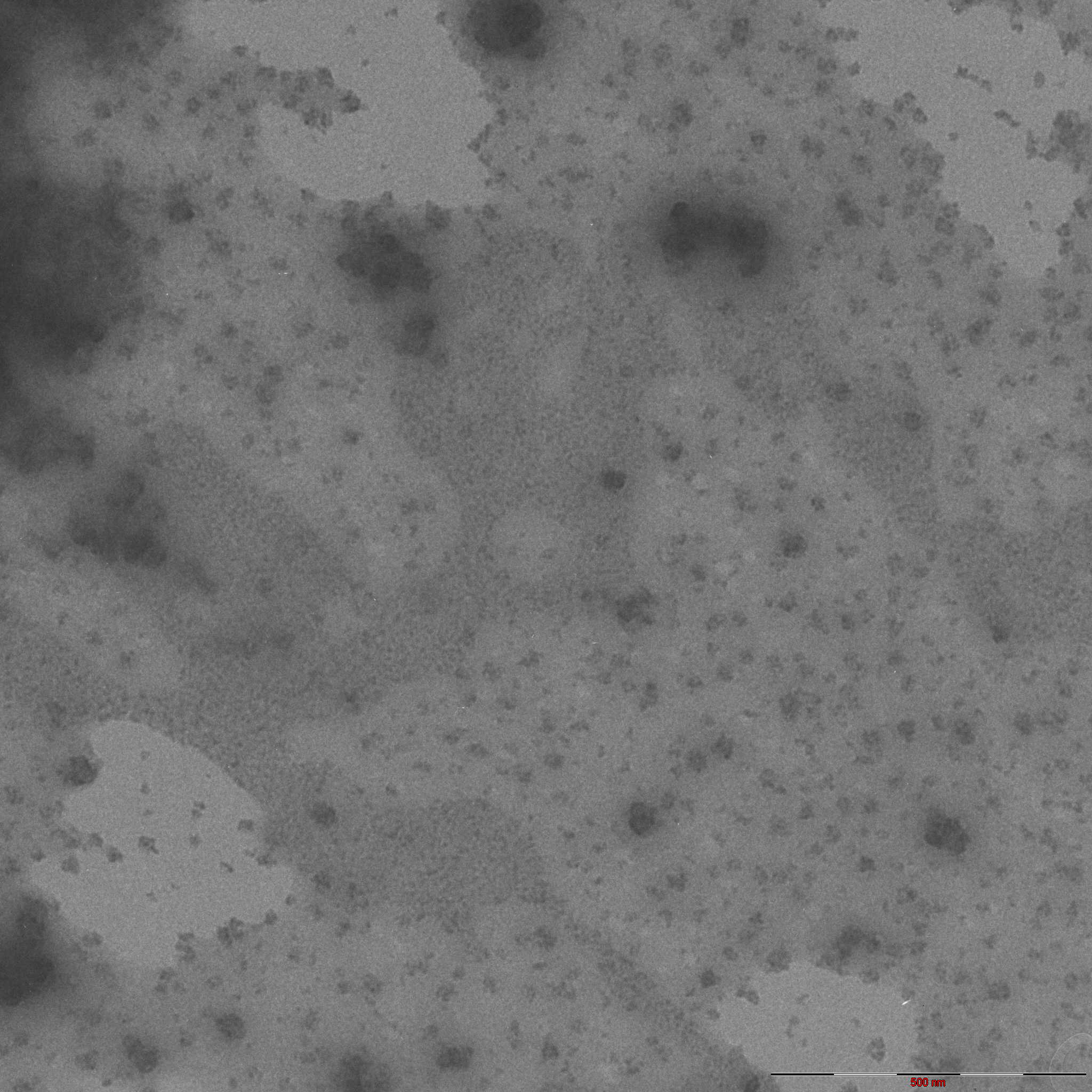

Supplement: Supplementary file 1 [file biomolecules-16-00891-s001.zip › TEM and IEM original images/Figure 1_IbpA PEG and ATP+30.jpg]

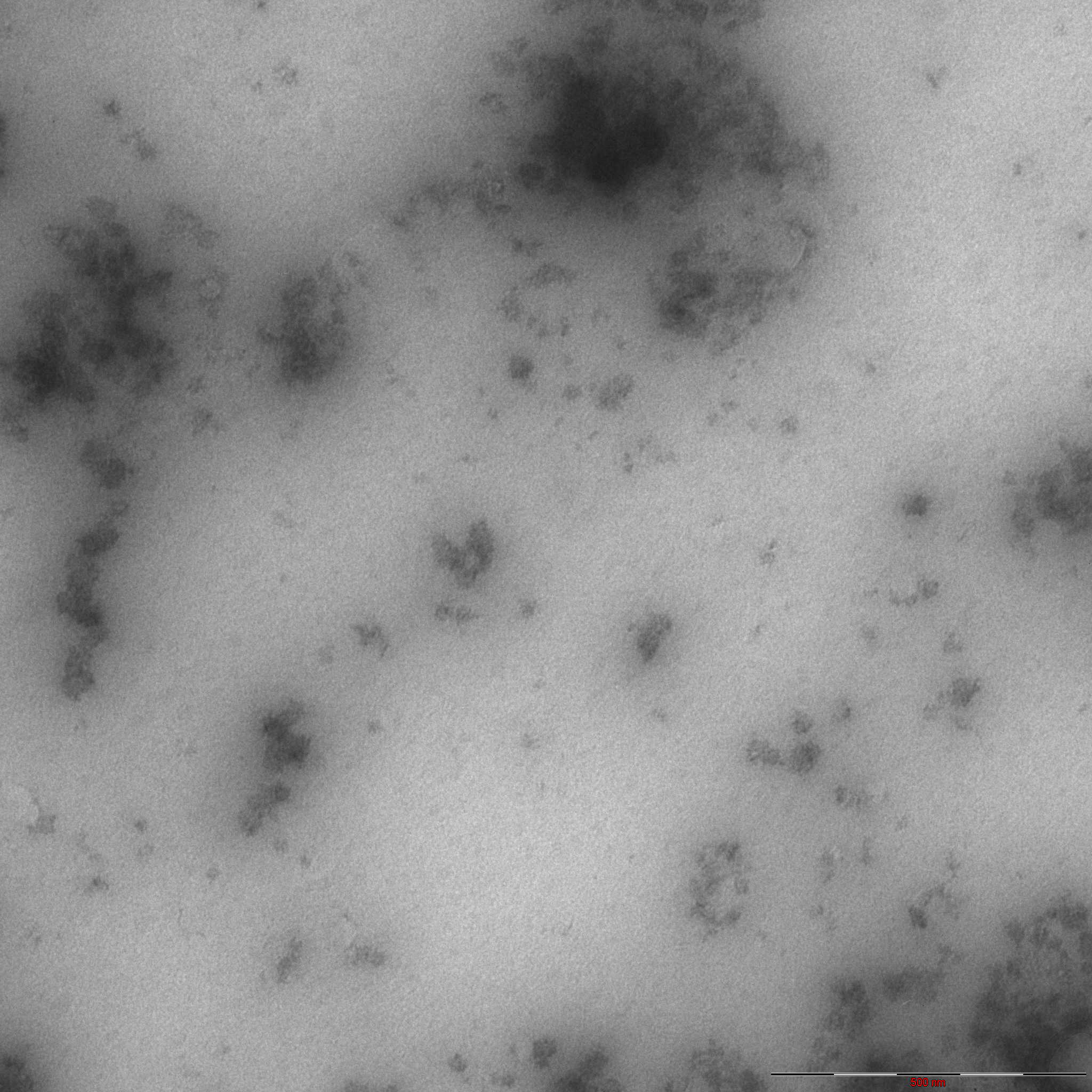

Supplement: Supplementary file 1 [file biomolecules-16-00891-s001.zip › TEM and IEM original images/Figure 1_IbpA PEG and ATP+37_02.jpg]

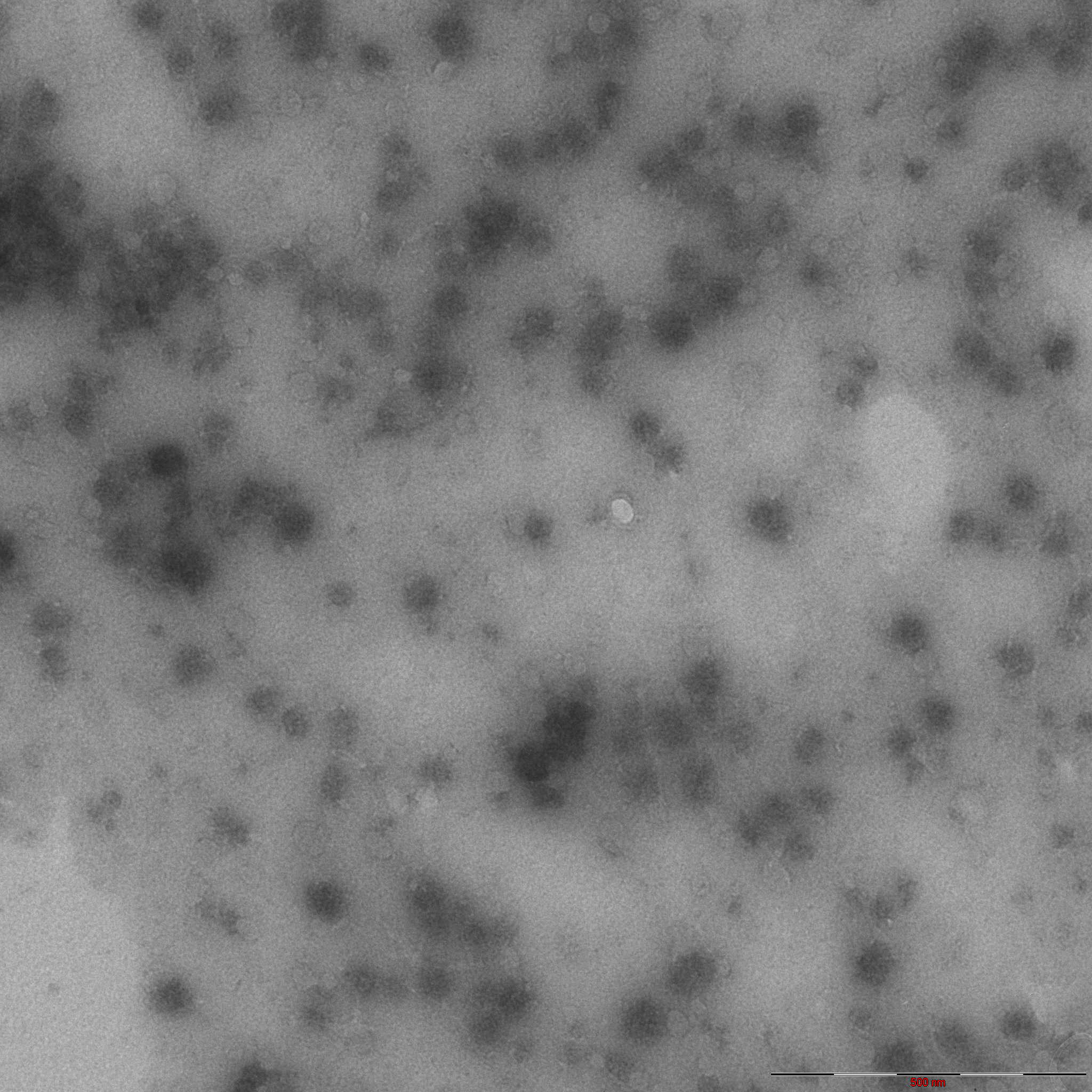

Supplement: Supplementary file 1 [file biomolecules-16-00891-s001.zip › TEM and IEM original images/Figure 1_IbpA PEG and ATP+42_10.jpg]

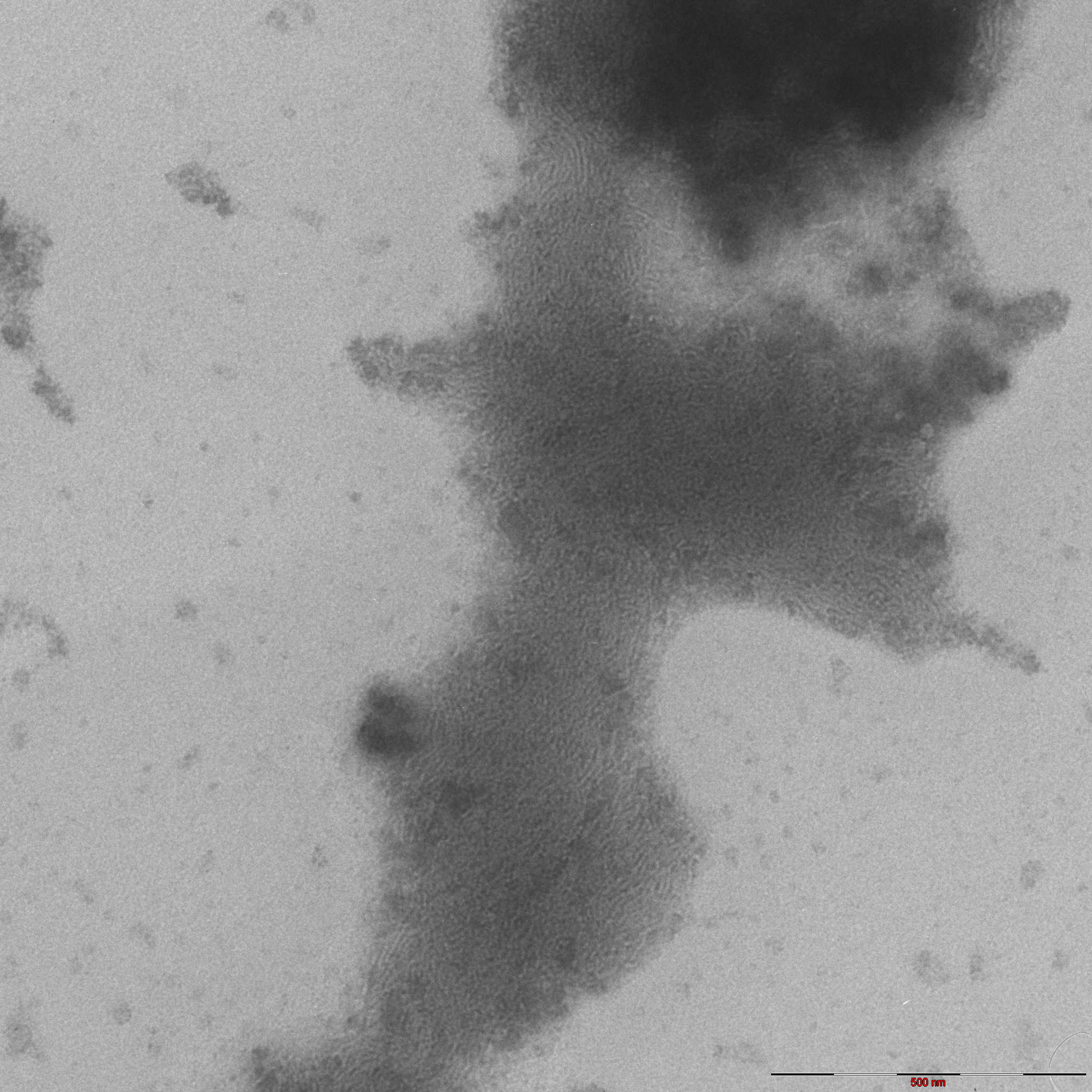

Supplement: Supplementary file 1 [file biomolecules-16-00891-s001.zip › TEM and IEM original images/Figure 1_IbpA PEG and ATP+4_05.jpg]

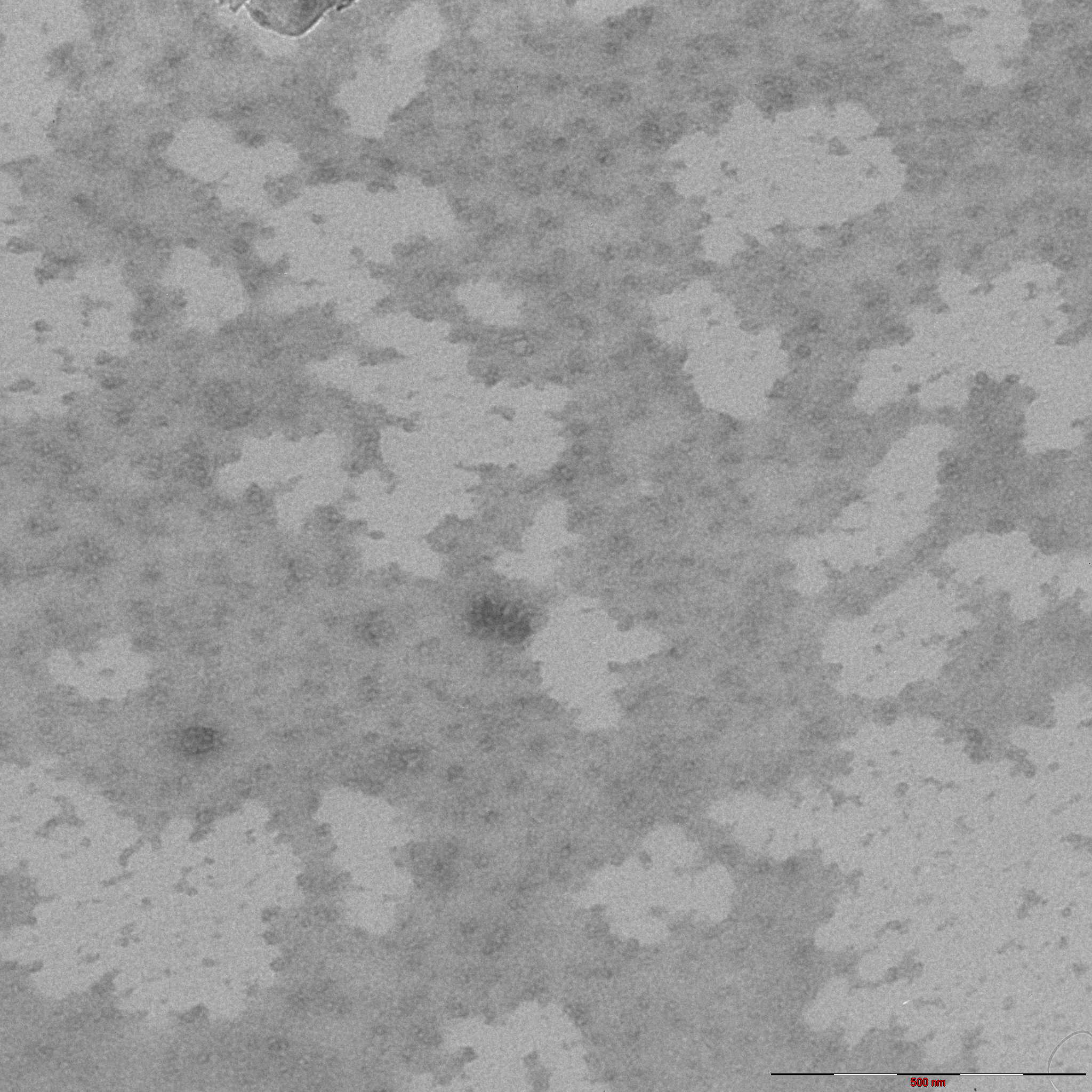

Supplement: Supplementary file 1 [file biomolecules-16-00891-s001.zip › TEM and IEM original images/Figure 1_IbpA PEG and GTP+30_04.jpg]

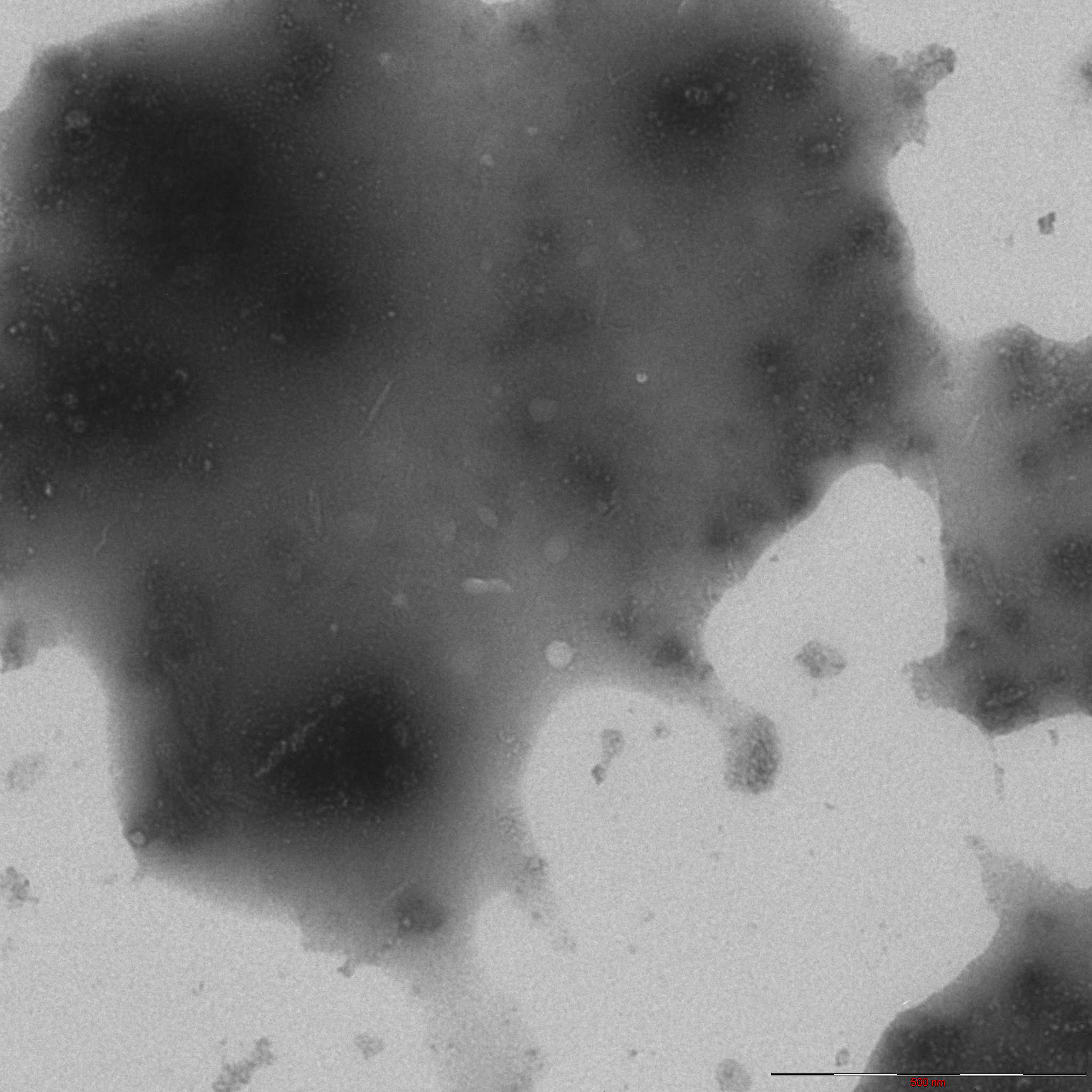

Supplement: Supplementary file 1 [file biomolecules-16-00891-s001.zip › TEM and IEM original images/Figure 1_IbpA PEG and GTP+37_04.jpg]

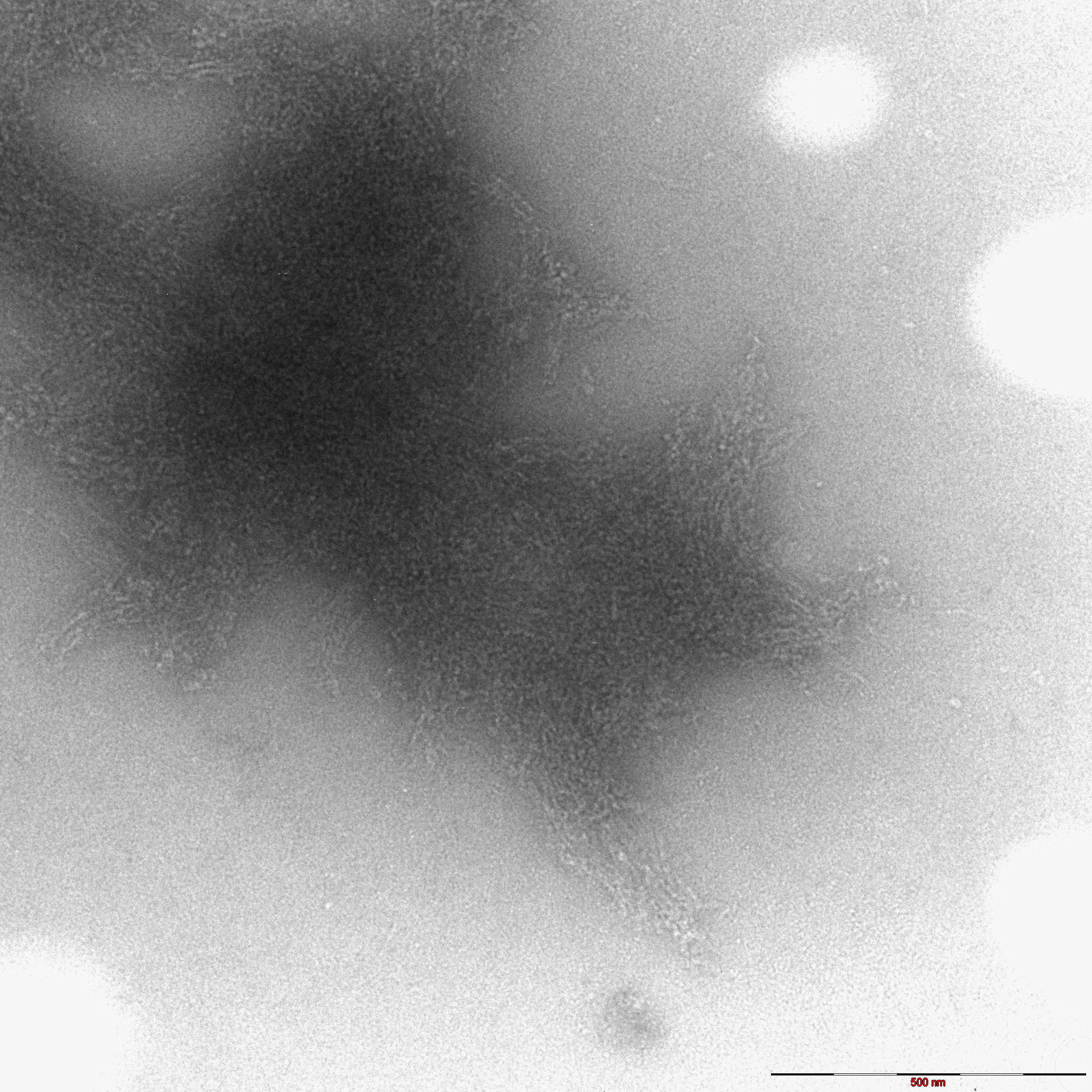

Supplement: Supplementary file 1 [file biomolecules-16-00891-s001.zip › TEM and IEM original images/Figure 1_IbpA PEG and GTP+4.jpg]

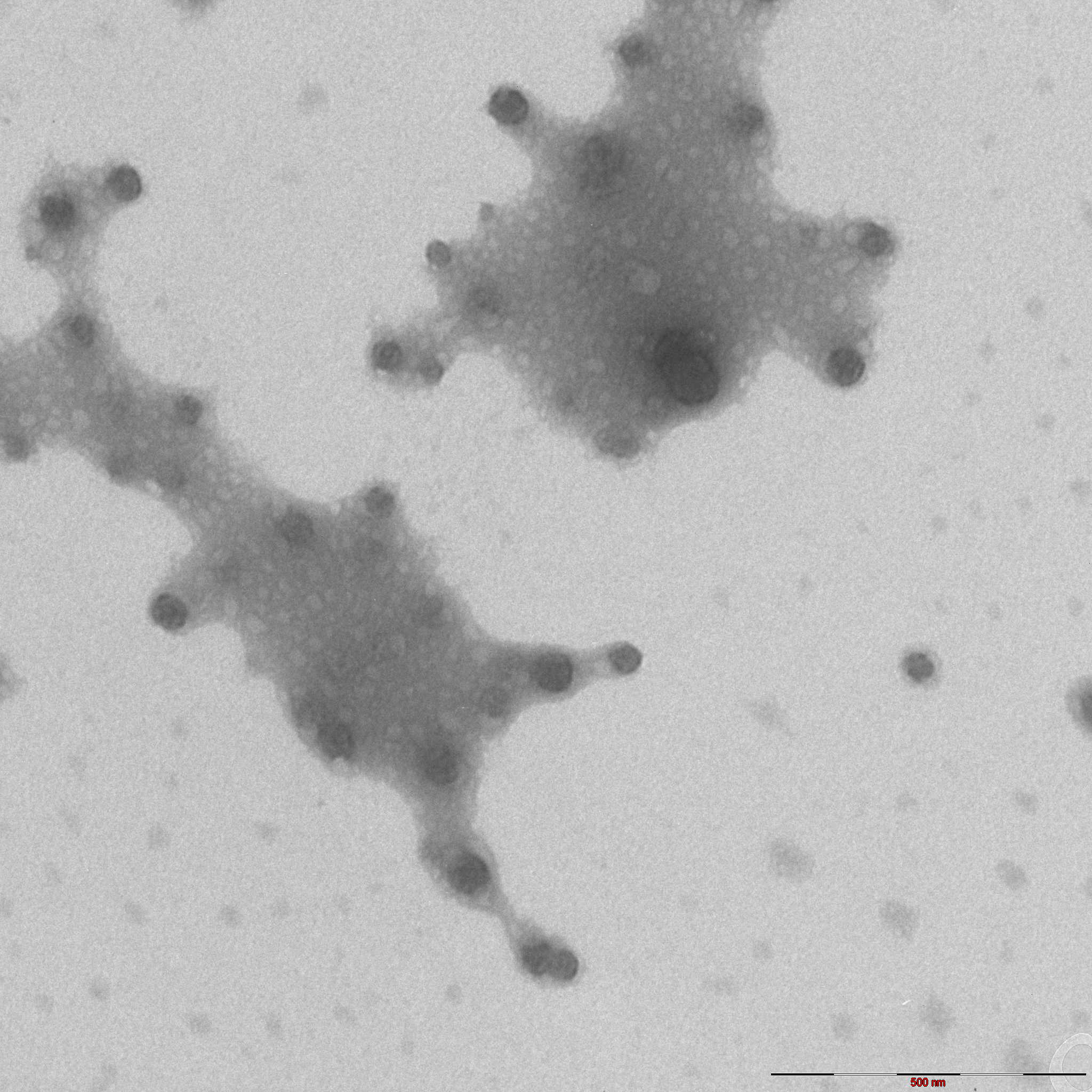

Supplement: Supplementary file 1 [file biomolecules-16-00891-s001.zip › TEM and IEM original images/Figure 1_IbpA PEG and GTP+42_10.jpg]

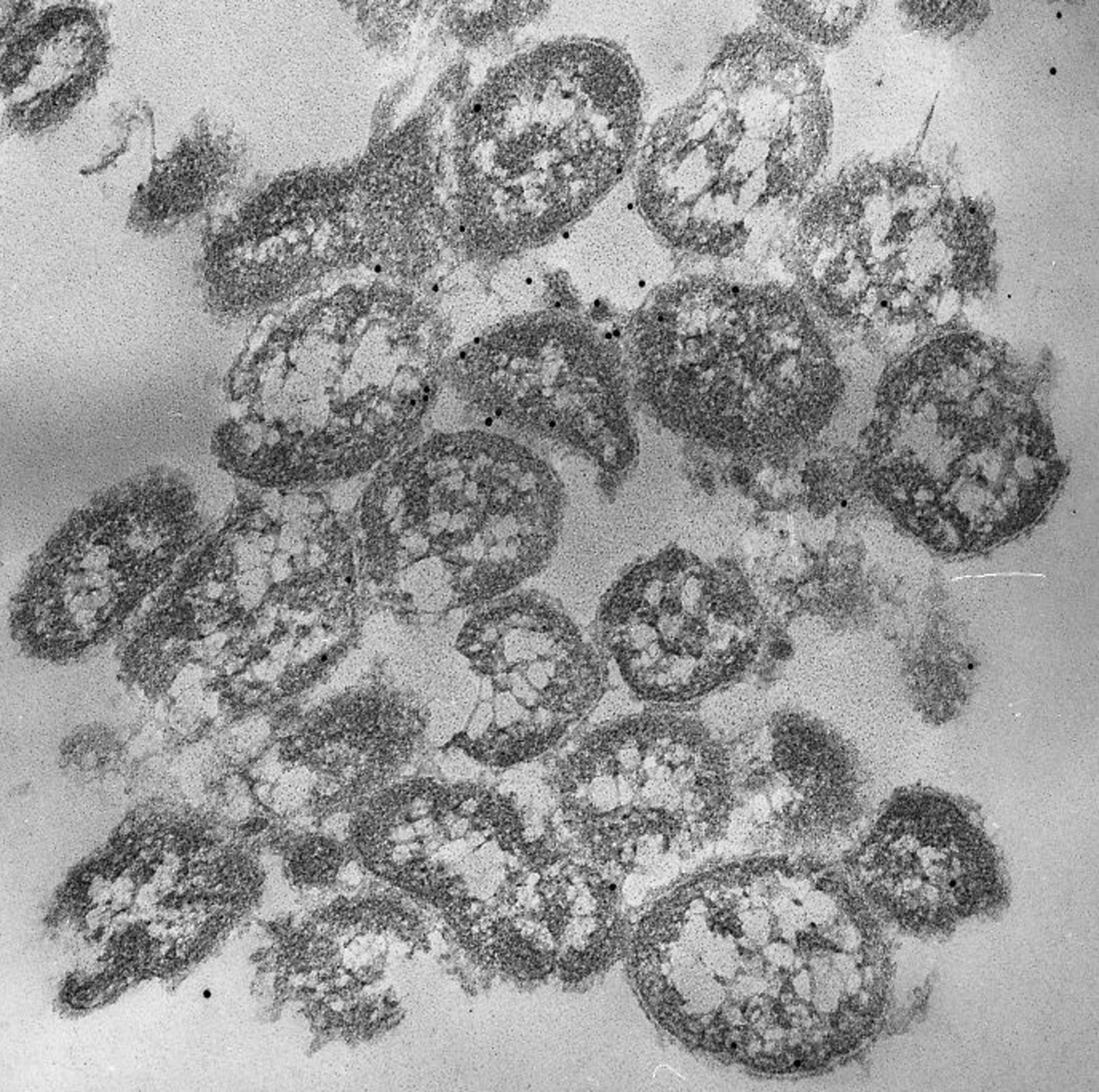

Supplement: Supplementary file 1 [file biomolecules-16-00891-s001.zip › TEM and IEM original images/Figure 2_IEM+37.jpg]

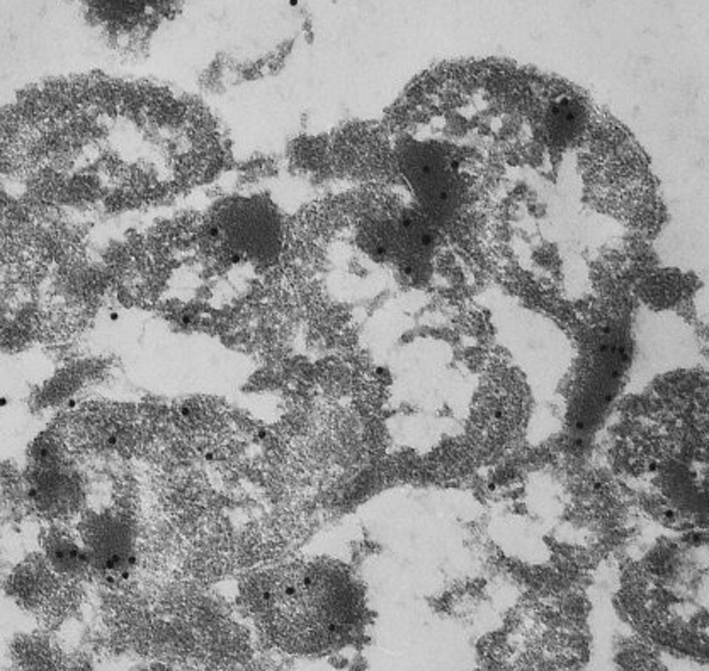

Supplement: Supplementary file 1 [file biomolecules-16-00891-s001.zip › TEM and IEM original images/Figure 2_IEM+42.jpg]

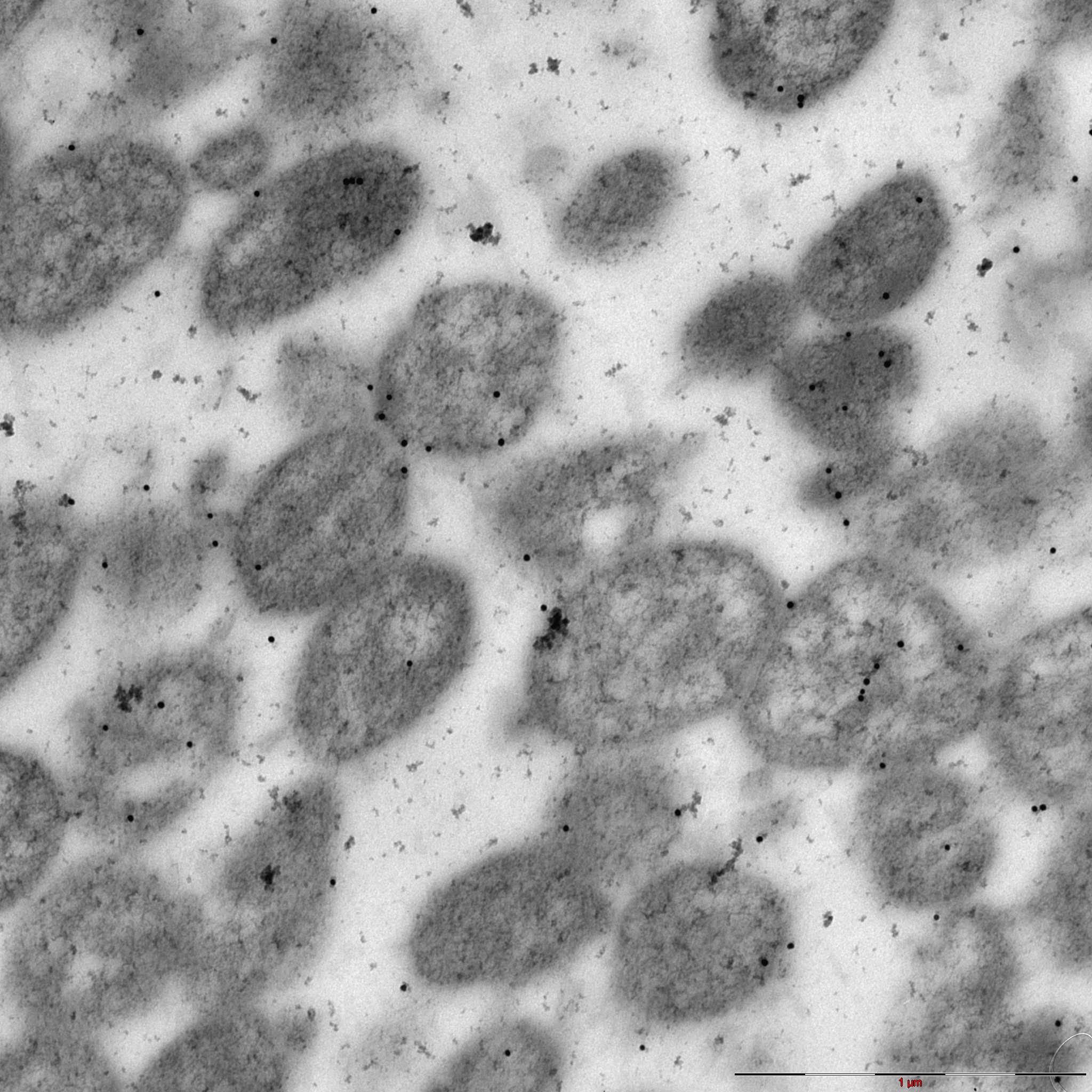

Supplement: Supplementary file 1 [file biomolecules-16-00891-s001.zip › TEM and IEM original images/Figure 3_IEM+4_01.jpg]

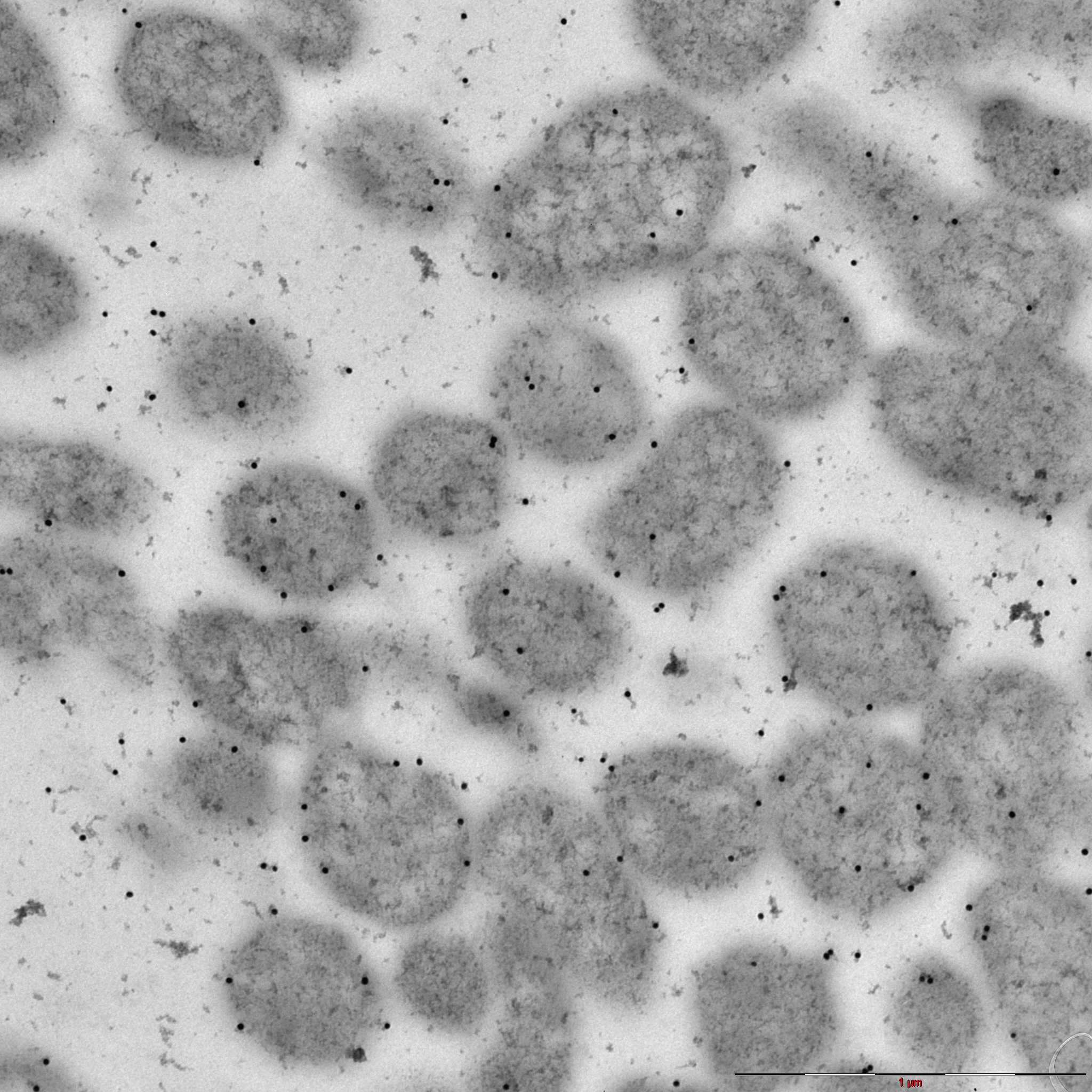

Supplement: Supplementary file 1 [file biomolecules-16-00891-s001.zip › TEM and IEM original images/Figure 3_IEM+4_02.jpg]

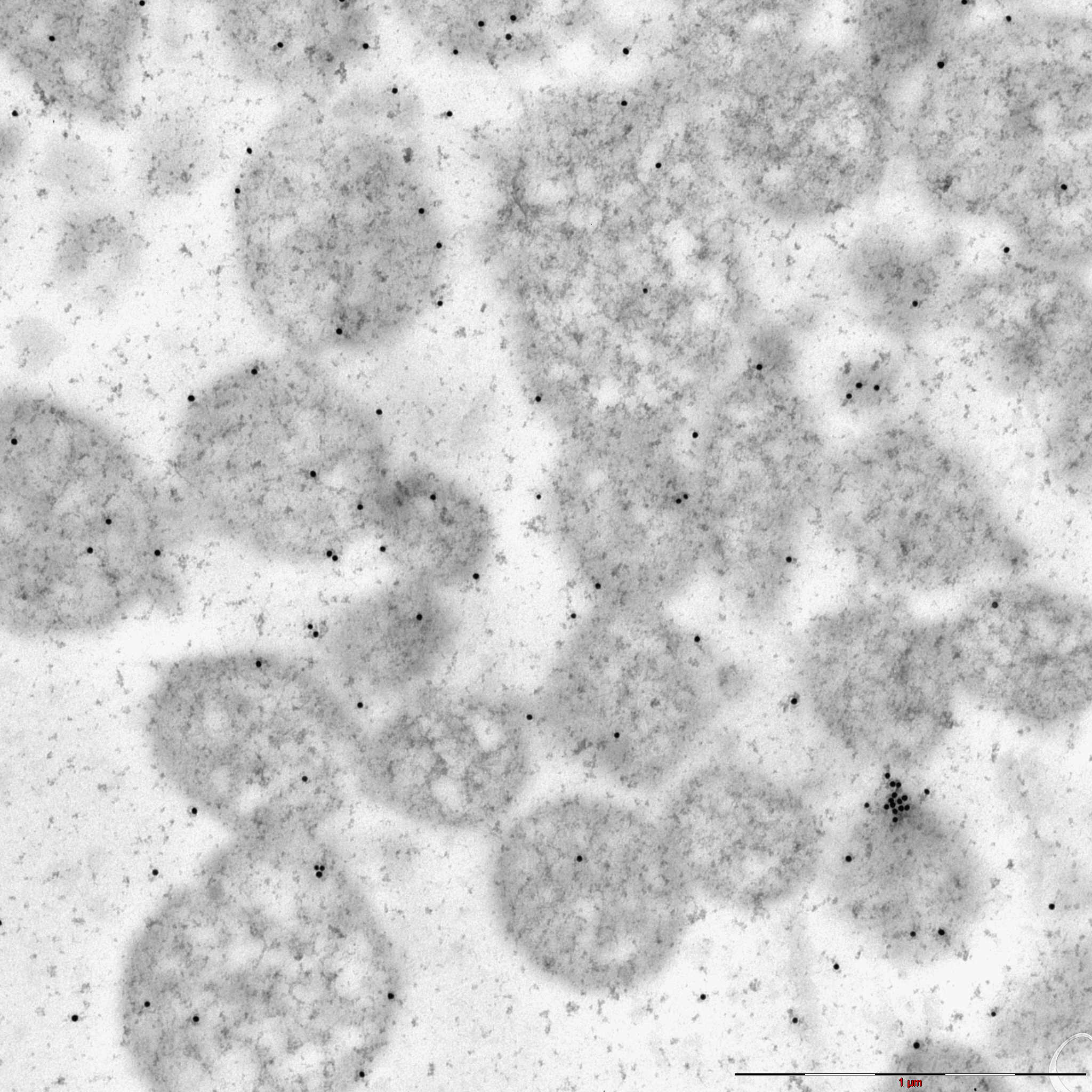

Supplement: Supplementary file 1 [file biomolecules-16-00891-s001.zip › TEM and IEM original images/Figure 3_IEM+4_03.jpg]

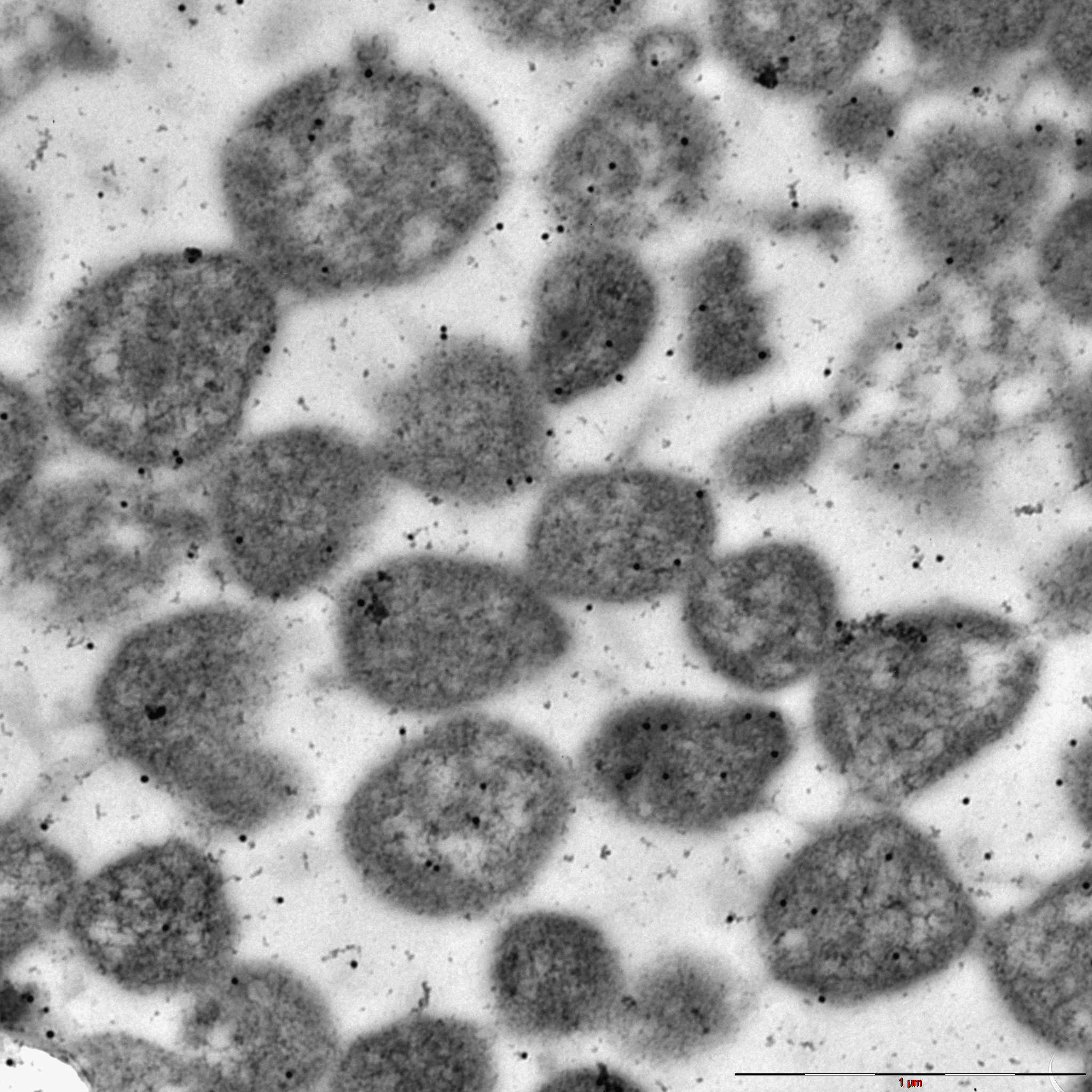

Supplement: Supplementary file 1 [file biomolecules-16-00891-s001.zip › TEM and IEM original images/Figure 3_IEM+4_04.jpg]

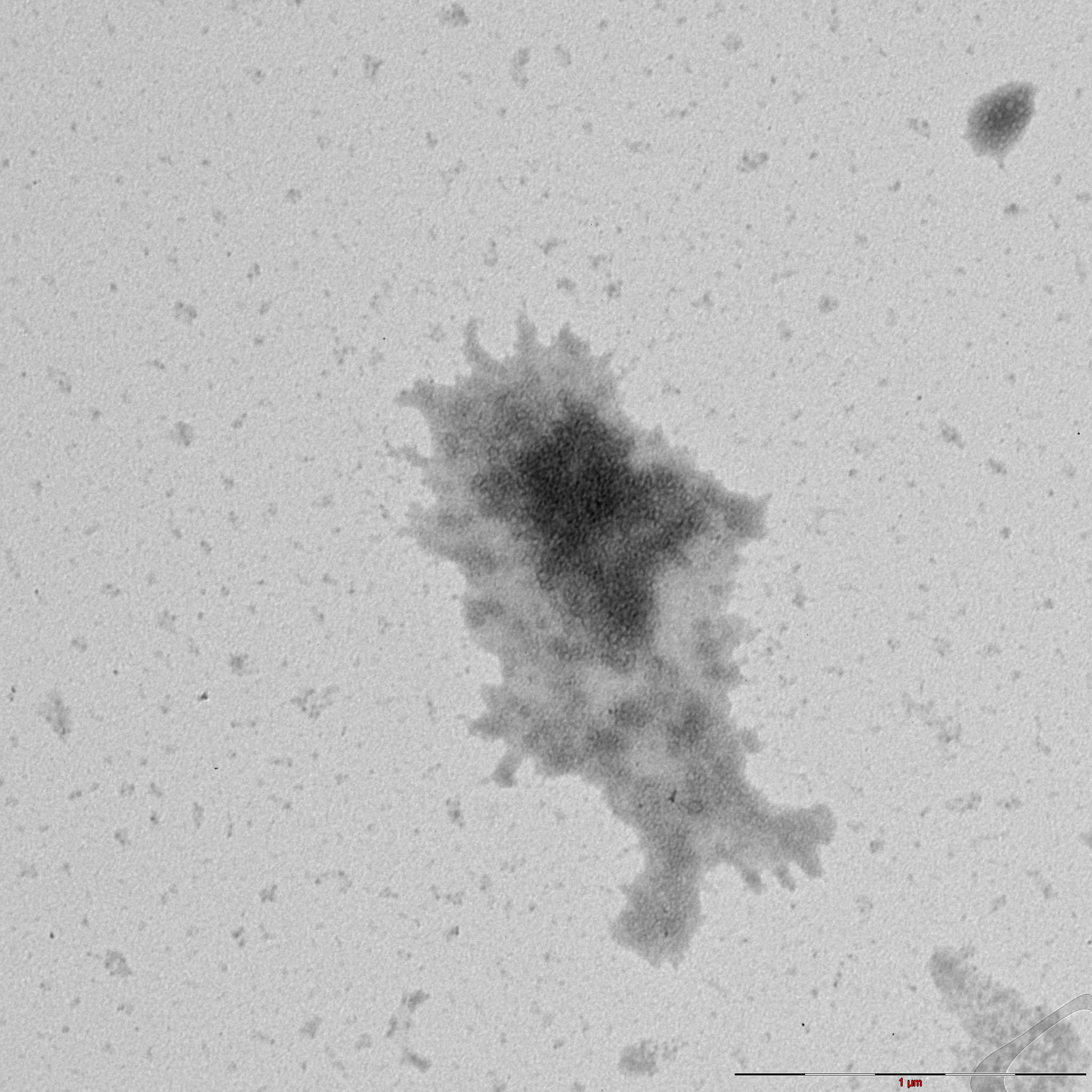

Supplement: Supplementary file 1 [file biomolecules-16-00891-s001.zip › TEM and IEM original images/Figure 4_I.jpg]

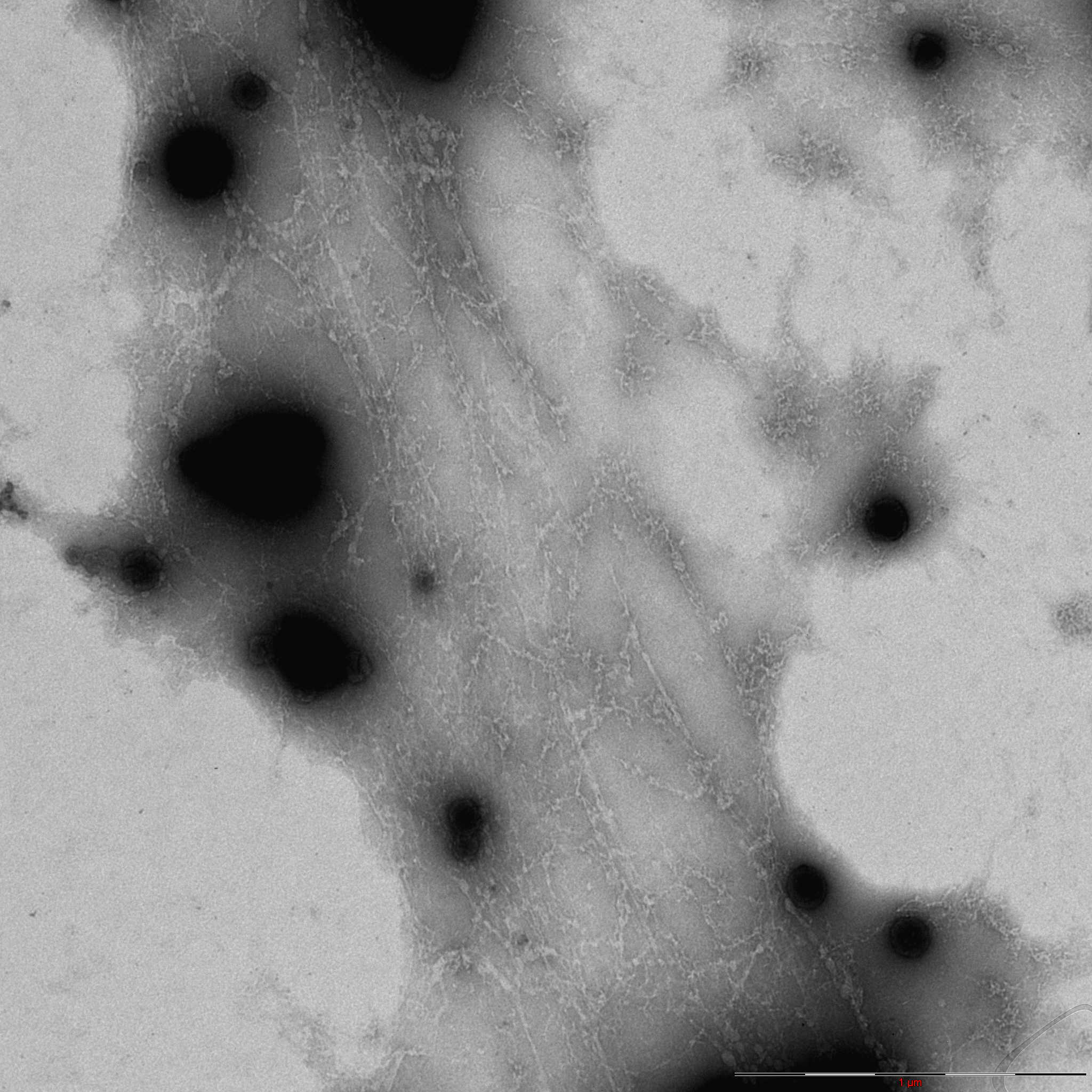

Supplement: Supplementary file 1 [file biomolecules-16-00891-s001.zip › TEM and IEM original images/Figure 4_II.jpg]

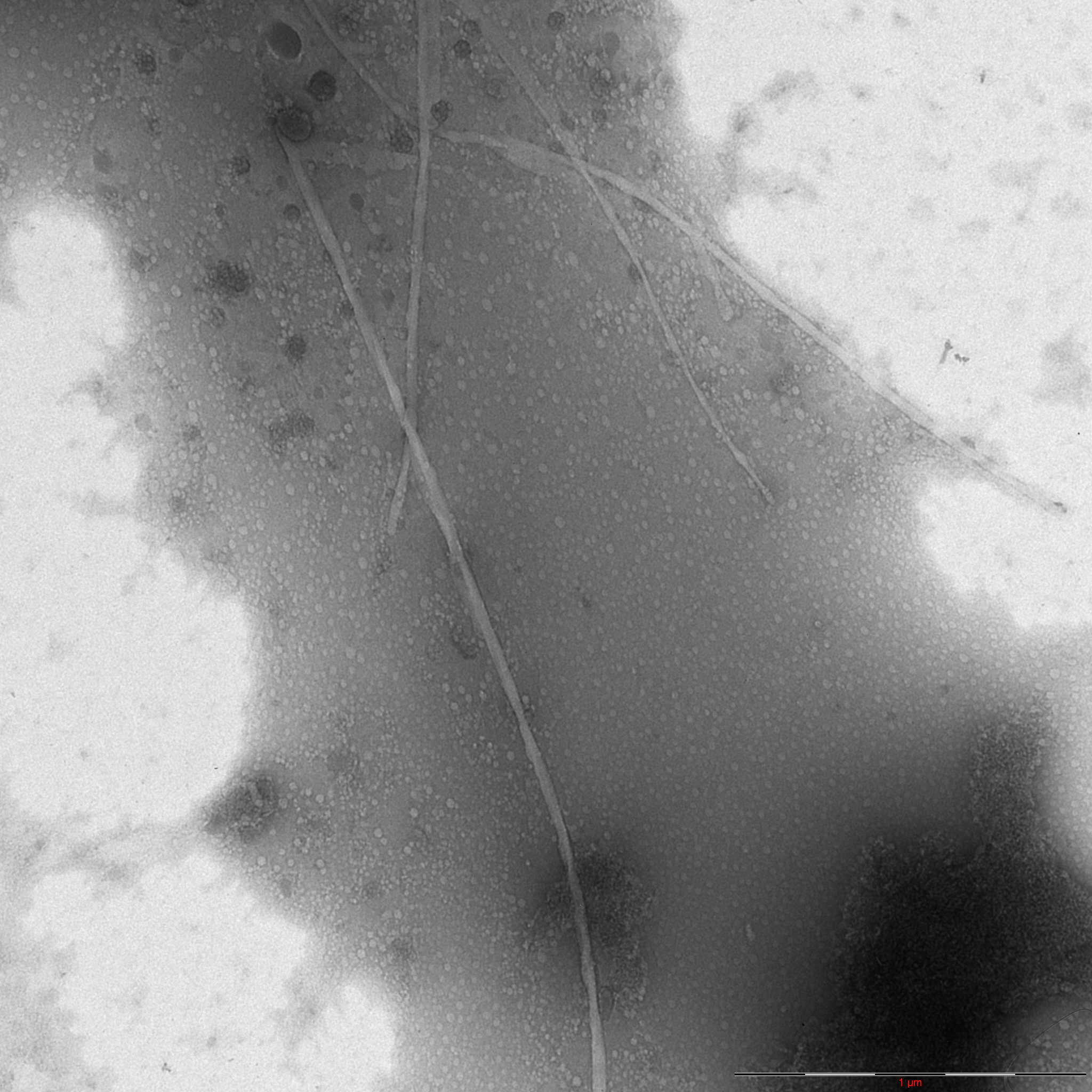

Supplement: Supplementary file 1 [file biomolecules-16-00891-s001.zip › TEM and IEM original images/Figure 4_III-insertion.jpg]

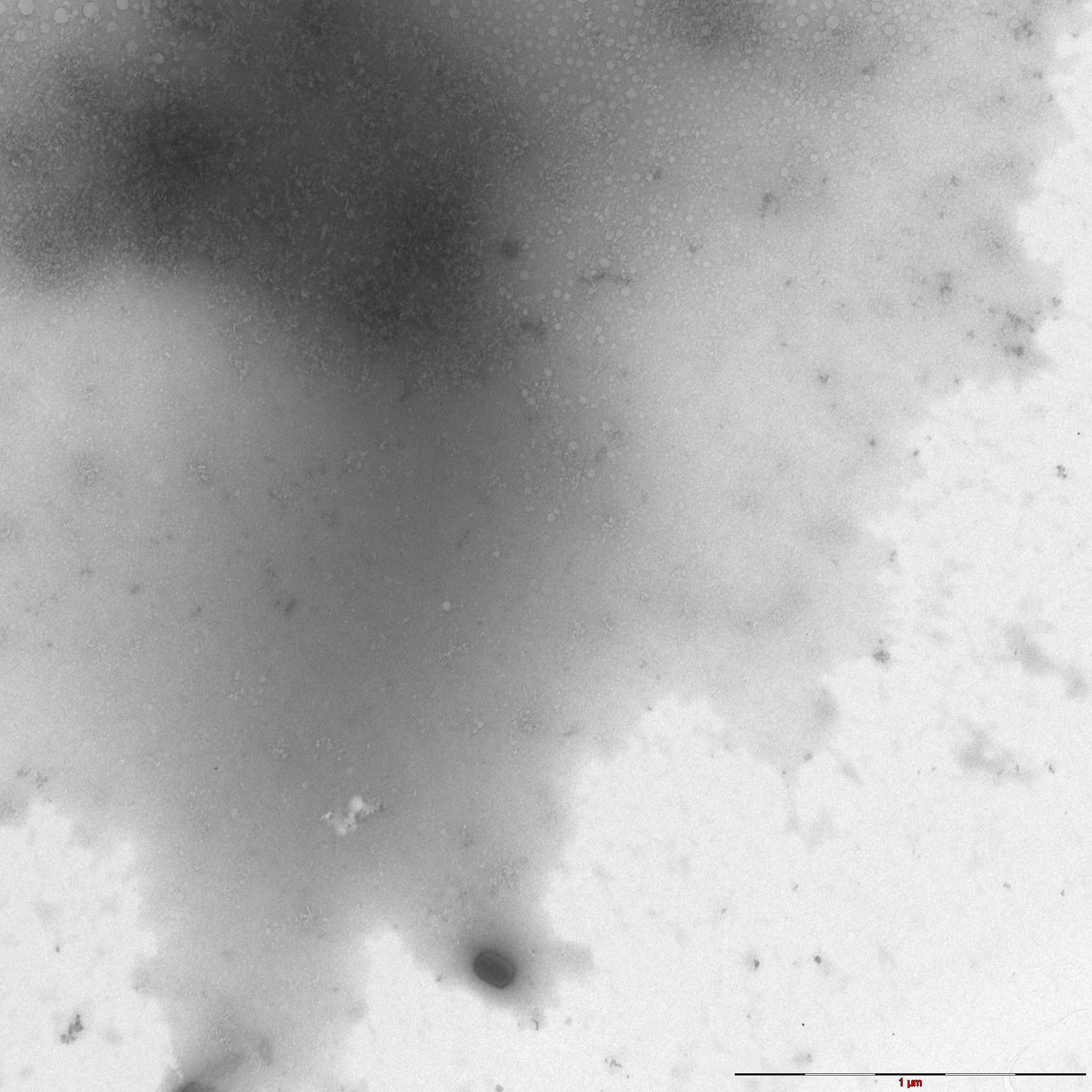

Supplement: Supplementary file 1 [file biomolecules-16-00891-s001.zip › TEM and IEM original images/Figure 4_III.jpg]

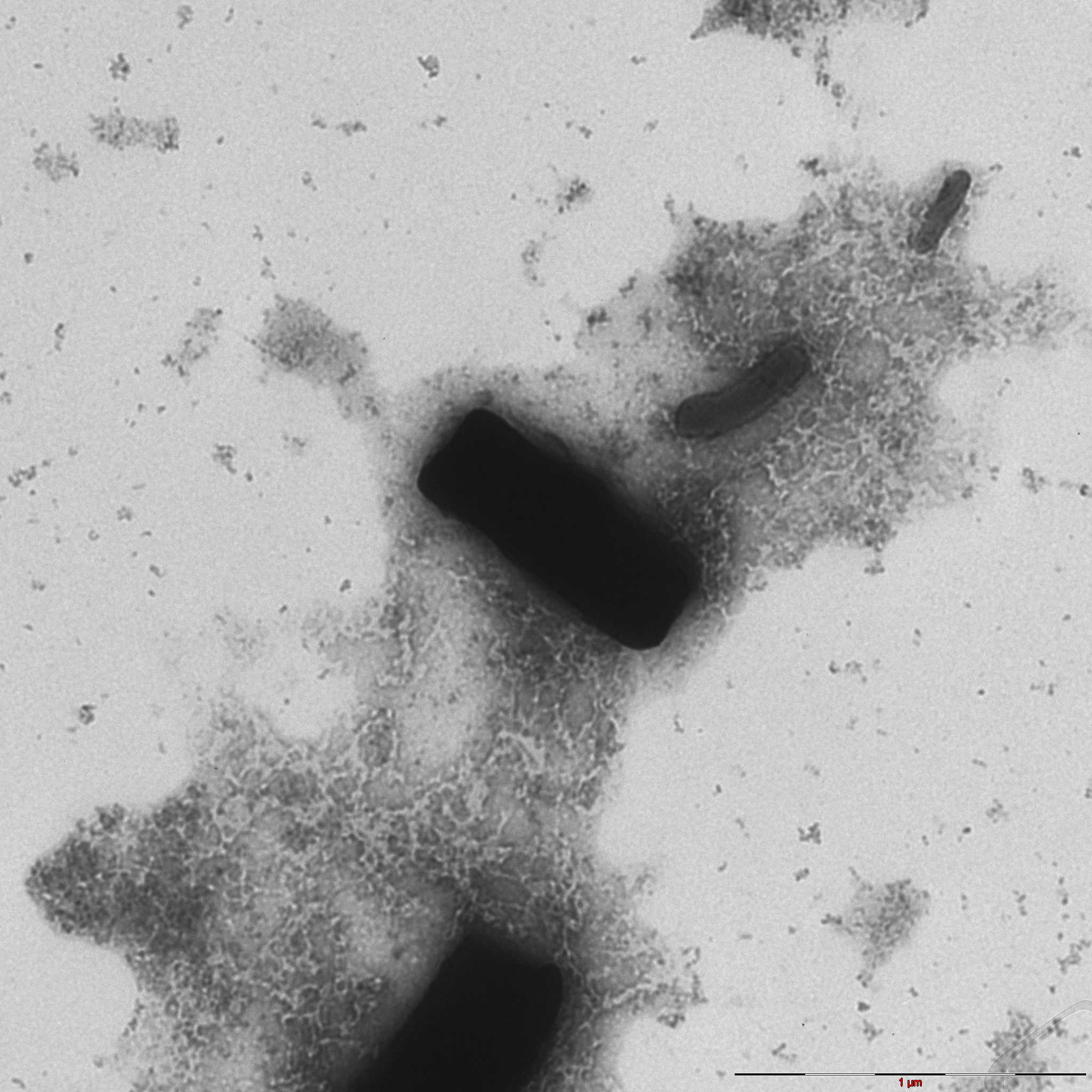

Supplement: Supplementary file 1 [file biomolecules-16-00891-s001.zip › TEM and IEM original images/Figure 4_IV-insertion.jpg]

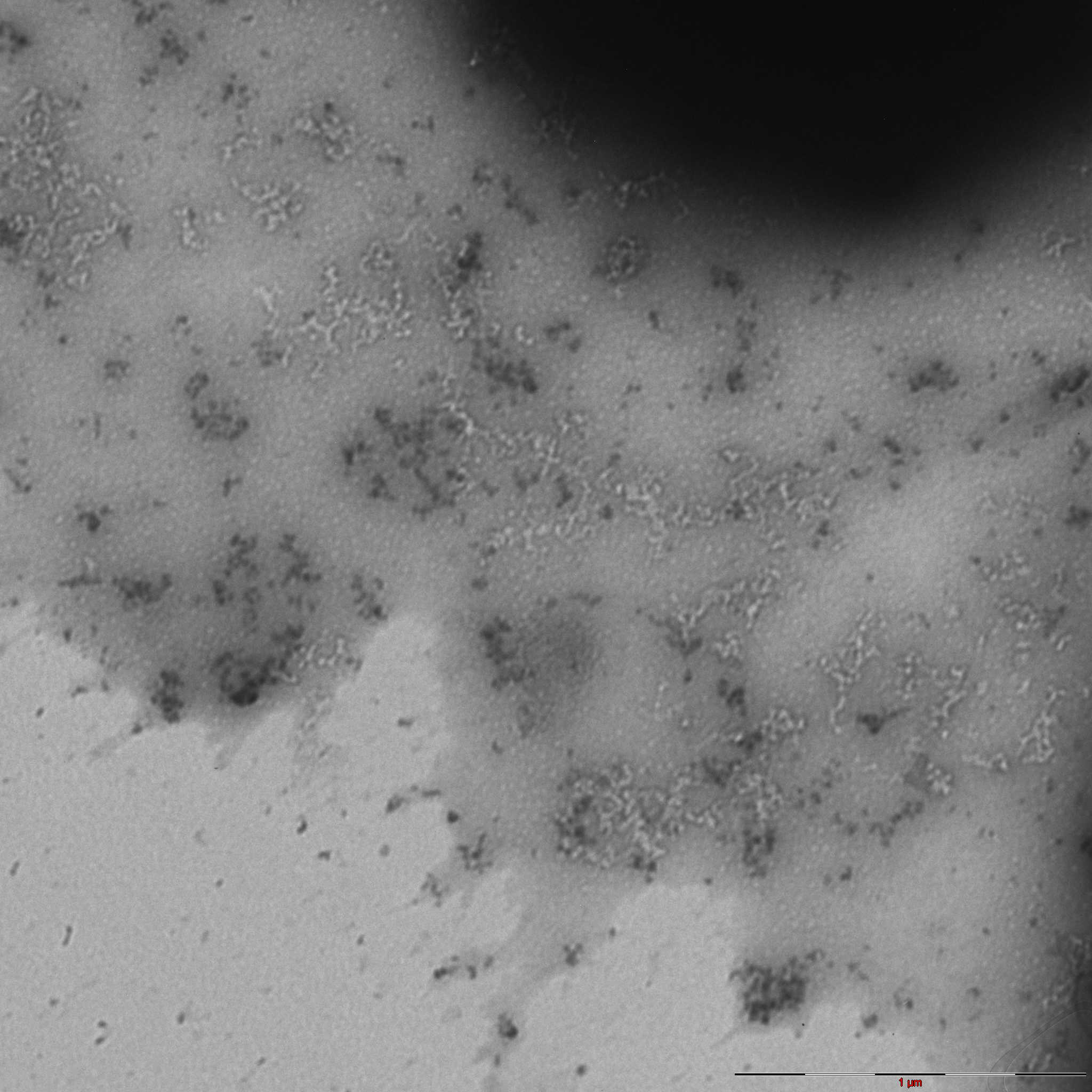

Supplement: Supplementary file 1 [file biomolecules-16-00891-s001.zip › TEM and IEM original images/Figure 4_IV.jpg]
